# Does Reiki Benefit Mental Health Symptoms Above Placebo?

**DOI:** 10.3389/fpsyg.2022.897312

**Published:** 2022-07-12

**Authors:** Sonia Zadro, Peta Stapleton

**Affiliations:** School of Psychology, Bond University, Gold Coast, QLD, Australia

**Keywords:** Reiki, placebo, mental health, anxiety, depression, stress, burnout, systematic review

## Abstract

**Background:**

Reiki is an energy healing technique or biofield therapy in which an attuned therapist places their hands on or near the client’s body and sends energy to the client to activate the body’s ability to heal itself and restore balance. It was developed in Japan at the end of the 19th century by Mikao Usui of Kyoto. Given the enormous international socioeconomic burden of mental health, inexpensive, safe, and evidenced-based treatments would be welcomed. Reiki is safe, inexpensive, and preliminary research suggests it may assist in treating a wide variety of illnesses. Given that Reiki is a biofield therapy, growing in use, and not yet accepted by the dominant biomedical paradigm, it is important to establish its effectiveness over placebo. This study aimed to examine Reiki’s effectiveness over placebo in treating symptoms of mental health and to explore parameters for its effectiveness.

**Method:**

A systematic review of randomized placebo-controlled trials (RPCTs) examining Reiki’s effectiveness in treating symptoms of mental health in adults was conducted through a systematic search of PubMed, PsycINFO, MEDLINE, CINAHL, Web of Science, Scopus, Embase, and ProQuest. Fourteen studies met the inclusion criteria, and risk of bias was assessed using Cochrane’s Revised ROB 2 assessment tool. This was followed by a grading of recommendations, assessment, development and evaluations (GRADE) assessment.

**Results:**

The evidence to date suggests that Reiki consistently demonstrates a greater therapeutic effect over placebo for some symptoms of mental health. The GRADE level of evidence is high for clinically relevant levels of stress and depression, moderate to high for clinically relevant levels of anxiety, low to moderate for normal levels of stress, and low to moderate for burnout, and low for normal levels of depression and anxiety.

**Conclusion:**

The results suggest that, Reiki may be more effective in treating some areas of mental health, than placebo, particularly if symptoms are clinically relevant. To date, there are a small number of studies in each area, therefore findings are inconclusive and, more RCTs controlling for placebo in Reiki research are needed. Most included studies were also assessed as having a risk of bias of some concern. Incorporating Reiki as a complementary treatment to mainstream psychotherapy for depression, stress, and anxiety may be appropriate.

**Systematic Review Registration:**

[https://www.crd.york.ac.uk/], identifier [CRD42020194311].

## Introduction

Complementary and alternative medicine (CAM) is a heterogeneous group of practices that are not part of orthodox medical care. They may include alternative medical systems such as Chinese medicine, mind-body interventions such as meditation, biologically based treatments such as herbs, body-based approaches such as chiropractic care (NIH, 2005), and biofield therapies which use the body’s energy field to promote therapeutic benefit (Guarneri and King, 2015). Reiki is considered a biofield therapy. It was developed in Japan at the end of the 19th century by Mikao Usui of Kyoto (Baldwin, 2020). It is a non-invasive treatment whereby the attuned practitioner gently places their hands on or close to the body of a client in a sequence of positions to promote the body’s ability to heal itself and restore balance (Anderson and Wolk-Weiss, 2008).

Worldwide, one billion people suffer from a mental health disorder (The Lancet Global, 2020), and mental illness accounts for half of all illnesses for people up to 45 years of age in wealthy countries (LayaRd, 2017). In 2010, mental health expenditure was estimated to cost the world economy USD$2.5 trillion a year because of poor health and reduced productivity, and this cost is expected to increase to USD$6 trillion by 2030 (The Lancet Global, 2020). Finding inexpensive, evidence-based, safe ways to reduce this burden would be welcomed. Reiki is deemed safe, inexpensive, and non-invasive and preliminary research suggests that it may be effective in treating many illnesses, including symptoms of mental health.

There is also evidence Reiki has therapeutic effects compared with placebo on non-human living systems. Studies on Reiki’s effects that best control for placebo are non-human studies where we would expect placebo not to be operating or be less influential. These include RPCT of Reiki’s influence on cell cultures, isolated cells, rats and dogs. Despite this expectation in all the following randomised controlled trials (RCTs), the placebo effect was still controlled for with a placebo Reiki group, and all produced significant therapeutic results (Baldwin and Schwartz, 2006; Baldwin et al., 2008; Mothersill et al., 2013; Kent et al., 2020; Pacheco et al., 2021). In these five studies, Reiki was more effective than placebo in the following areas: reducing noise-induced microvascular damage in rats (Baldwin and Schwartz, 2006), improving heart rate homeostasis in rats (Baldwin et al., 2008), increasing survival of directly irradiated cells (Mothersill et al., 2013), increasing photon emission of intervertebral cells in mice and increasing collagen 11 and aggrecan in mice (Kent et al., 2020), and reducing postoperative pain in female dogs undergoing elective minimally invasive ovariohysterectomy (Pacheco et al., 2021). While the studies do not prove that Reiki is beneficial over placebo in humans, they provide evidence that Reiki can be beneficial over placebo in non-human living systems.

Complementary and alternative medicine (CAM) and Reiki are also becoming frequently used and more accepted (Lepine, 2018), with reported rates of CAM use at 69% in Australia (Xue et al., 2007), 76% in Singapore, 76% in Japan, and 75% in South Korea (Wardle et al., 2018). According to the Center for Reiki Research (Lepine, 2018), 60 hospitals and clinics offer Reiki treatments in the United States (USA), and eight other clinics offer it in other countries. Despite the growing use, it is important to keep in mind that Reiki is a biofield therapy (B.T.) belonging to the paradigm of biofield science, which has not been fully established or accepted by the mainstream biomedical paradigm. The biofield is believed to be an organizing energy field of any living system that regulates and helps maintain the biological system (Rubik et al., 2015). This notion is a shift from the mechanical chemistry-based view of the current dominant biomedical paradigm to an information-based view where the biofield is a multi-level organizational concept where information flows within and between various levels of an organism (Rubik et al., 2015). Western biomedicine routinely examines electrical fields from the heart, known as electrocardiograms (ECG), and the brain, known as electroencephalograms (EEG). Biophysics provides evidence that endogenous electromagnetic and other energy fields influence tissue development, tissue repair, and other processes. Energy medicine purports that sending low-level signals to the body can help it heal, including energy healing interventions and bio-electromagnetic device-based therapies (Rubik et al., 2015). However, there are several sociological and paradigmatic oppositions to biofield science as follows (Hufford et al., 2015):

(1) Although research is emerging on causal factors to explain the biofield, such as electromagnetic field properties of electricity, magnetism, sound, and pH, they are not well-understood (Kent et al., 2020) and therefore viewed as unscientific (Hufford et al., 2015).
(2) Some scientists believe that biofield science is incongruent and, therefore, a challenge to the current paradigm.
(3) Subtle energies are often viewed as central to biofield healing. The notion that subtle energies exist is not accepted by conventional science so they are often viewed as psuedoscience.
(4) The idea of a biofield is often associated with a life force or vitalism, that is, the historical notion of a force behind consciousness. This was firmly rejected by modern medicine and still is.

The above concerns point to a need for scientific inquiry into whether Reiki is more effective then placebo, particularly in the much needed area of mental health. This systematic review aims to determine the level of evidence for Reiki’s effectiveness over placebo and explore the parameters used when and if it is more effective.

## Method

Reiki’s effectiveness over placebo in treating mental health was assessed by a systematic review (SR) of RCTs in which a placebo-Reiki comparison group was included. In most randomized placebo-controlled trials (RPCTs), of Reiki, an untrained Reiki practitioner with no knowledge of Reiki is taught to mimic the hand positions of the trained Reiki practitioner to control for expectancy effects on participants. The participants are blind to whether they are receiving real or sham Reiki. As much as possible, all other conditions, room, lighting, time, sound, etc., are kept the same as in the Reiki condition. To control for client expectancy effects, creating a placebo-Reiki group goes beyond standard RCT protocols and provides more rigorous evidence.

This study only focused on studies that compared hands-on Reiki to a placebo Reiki group or controlled for placebo in some other way. It did not include distant Reiki because it was thought that the causal mechanisms operating might be different for distant Reiki applications. Also, in the hands-on placebo Reiki condition, while participants are not receiving Reiki, they often receive touch unless Reiki is delivered close to but not touching the body. Several studies suggest that the use of therapeutic touch alone has benefits (Gagne and Toye, 1994; Hawranik et al., 2008; So et al., 2008; Ana Cristina et al., 2016). As such, studies of hands-on placebo Reiki involving touch may be examining the benefits of Reiki over therapeutic touch and placebo.

### Considerations in Studying Reiki

In examining whether Reiki has a therapeutic effect over placebo, some considerations are as follows to achieve a therapeutic effect:

(1) How long and how many applications of Reiki are needed, and is the dose varied for different conditions?
(2) What kinds of conditions does Reiki provide a therapeutic effect for?
(3) If Reiki is therapeutic, how long do the benefits last?
(4) Do the length of time and number of applications vary according to the level of training and experience of the practitioner?
(5) If effective, do the benefits always occur during treatment, or could they occur after treatment?

### Inclusion Criteria for Review

While several reviews of randomized RCTs of Reiki’s effectiveness have been conducted (Vitale, 2007; Lee et al., 2008; VanderVaart et al., 2009; Joyce and Herbison, 2015; Zimpel et al., 2020; Morero et al., 2021), only one focused solely on studies controlling for placebo (McManus, 2017); however, it did not conform to the systematic review (SR) methodology. The present study aimed to provide an up-to-date review of RPCTs on the influence of Reiki on mental health symptoms in adults that conformed to the SR methodology and included an assessment of the risk of bias (ROB) and the GRADE criteria. It also included a broader range of inclusion criteria than McManus (2017) as follows:

P: Adults healthy or unhealthy 18 years or older, with or without a formal diagnosis of a mental health condition.

I: Hands-on (touching or near the body) Reiki as a treatment intervention regardless of frequency, duration, or level of practitioner training.

C: A Placebo or Sham comparison group where Reiki is mimicked and/or the receiver did not know whether they received Reiki or not. Other comparison groups may also be included.

O: Standardized, valid, and reliable outcome measures of mental health symptoms and statistical analysis of results.

Published and unpublished studies were included, but only those translated into English.

Despite other SRs of Reiki not meeting the inclusion criteria of a placebo group, there are differences in other Reiki SRs’ worth noting. For instance, a Cochrane SR (Zimpel et al., 2020) of “Complementary and alternative therapies for post-cesarean pain” included a review of Reiki plus analgesia vs. analgesia. This only included 2 RCTs (Midilli and Eser, 2015; Midilli and Gunduzoglu, 2016), one of which did not have a placebo group (Midilli and Eser, 2015). The limited number of studies may have been due to four databases being searched rather than the eight searched in this SR.

Another SR by Joyce and Herbison (2015) excluded studies that were included in this SR because they did not meet the criteria for being anxious or depressed (Dressen and Singg, 1998; Shiflett et al., 2002; Mackay et al., 2004; Shore, 2004; Bowden et al., 2010). These studies still used *outcome measures of mental health* (met PICO), so they were included in this SR. Also, when baseline pre-treatment mean scores were compared to accepted clinical cutoffs, some studies that Joyce and Herbison (2015) excluded were in the clinical range (Dressen and Singg, 1998; Shore, 2004), and one study she included was in the clinical range for stress but not anxiety and depression (Bowden et al., 2011). These clinical discrepancies are highlighted in the results of this SR.

### Research Questions

1. Does Reiki consistently demonstrate a therapeutic effect above placebo for mental health symptoms?
2. If so, what variables appear to contribute to this therapeutic effect, i.e., duration and frequency of treatment and level of wellness?

### Search Method

Randomized controlled placebo trials were researched on PubMed, PsycINFO, MEDLINE, CINAHL, Web of Science, Scopus, Embase, and ProQuest with the keywords “Reiki,” “sham,” “placebo,” “mock,” and “comparison treatment.” Supplementary Appendix A lists the search terms used for each database. Reference lists of identified studies were scanned to search for additional studies. A total of 319 duplicates were removed. One hundred and eighty-nine records were screened, and of these, 150 were excluded and 39 were sought for retrieval. Two reports were not retrieved and 37 were assessed for eligibility. Two studies were in French, 4 used distant Reiki, and 4, on closer inspection, had significant design flaws due to non-randomization (Bessa et al., 2017), missing validated post-test outcome measures (Mauro, 2001), no statistical tests of significance (Salach, 2006), no validated outcome measures (Bourque et al., 2012), and a focus on senders of Reiki rather than recipients (Barnett, 2005). Finally, 12 studies with no outcome measures of mental health were excluded and 14 studies remained. The search was also conducted by an independent assessor. The PRISMA flow chart detailing search findings can be seen in Supplementary PRISMA Flow Diagram 1. The PRISMA Checklist can be found in Supplementary Appendix B. The PRISMA Abstract Checklist can be found in Supplementary Appendix C.

The SR used the recently revised Cochrane Risk of Bias Tool for randomized controlled trials (ROB 2). The suggested algorithms for judging risk of bias (ROB) were used as a guide to help maintain objectivity (Sterne et al., 2019). The ROB 2 uses a detailed template and an algorithm for judging ROB arising from each of the following areas: randomization, assignment to intervention, adhering to intervention, missing outcome data, measurement in the outcome, and selection of the reported result. ROB from period and carryover effects in crossover trials was applied if applicable. All ROB 2s were independently reviewed, and discrepancies were resolved. Full ROB 2 assessments for each study are available at https://cloudstor.aarnet.edu.au/plus/s/qI4RRkw4Ys8MFKr and effect size calculations at https://cloudstor.aarnet.edu.au/plus/s/uCKeNLEjGLo5jId. A summary of key information is presented in the results section Table 1 (Summary of Main Findings). Extended information on all studies is presented in Supplementary Table 1 (Extended summary of findings) of the Supplementary Material. Effect sizes were calculated for all RPCTs using an effect size calculator (Wilson, 2017), and the results were randomly checked by calculating Cohen’s d from *t*-tests (Thalheimer and Cook, 2002). For four studies, effect sizes could not be calculated because of insufficient published data (Shiflett et al., 2002; Rosada et al., 2015; Erdogan and Cinar, 2016; Çinar et al., 2022).

**TABLE 1:** Summary of main findings.

Study	Results Between groups *P*-value and effect size *d* = 0.2 (Small) 0.5 (Medium) 0.8 (Large).	ROB 2	Critique
**Clinical anxiety GRADE: moderate to high**			
Çinar et al. (2022) Fibromyalgia	**Post 4th trt Reiki sig lower than placebo for state anxiety (*p* = 0.005) and trait anxiety (*p* = 0.003).**	Some concerns	-No prespecified analysis plan was found. -1 exclusion criteria may have introduced bias. -Reiki was done by “an experimenter” but unclear if it was an author.

Baldwin et al. (2017) Knee Replacement Surgery	No between group comparisons made due to small sample size of controls. **Within grp Reiki sig *p* = 0.004 and Placebo NS.** ***d* (Reiki over placebo) = 0.93**	Some concerns	-If the Reiki size was better matched, placebo Reiki may not have performed as well. -Blinding was assessed as successful. -No pre-specified analysis plan was found.

Dressen and Singg (1998) Chronically ill and in pain.	**Reiki sig > placebo (no *p*-value)** **State Anx *p* = 0.0001 (pre/post) *d* = 1.36 > placebo.** **Trait Anx *p* = 0.0001 (pre/post) *d* = 1.07 > placebo.**	Some concerns	-Unstated if assessors were blind. -No pre-specified analysis plan was found. -Follow-up Reiki scores were not compared to other groups

**Anxiety: normal range GRADE: low**			
Bowden et al. (2011) Mood and wellbeing in High vs. Low Mood	HADS a*nxiety* baselines not given Reiki not sig > Placebo. DASS Anxiety (high mood) baseline borderline normal/low (7) Anxiety DASS High Mood *p* = 0.084 Low Mood *p* > 0.05	Some concerns	-Very small sample decreased power. -Experimenter administered Reiki however well blinded and participant blinding was assessed as successful.

Bowden et al. (2010) Well-being and salivary cortisol in healthy psych undergraduates.	Anxiety subscale DASS *p* = 0.295 *d* = 0.17 (L)	Some concerns	-Author administered Reiki but unlikely influence as sat behind a blindfolded SS with no touch (blinding successful *p* < 0.05) -No pre-specified plan. -Dropouts 14%

Thornton (1991) Female Nurses Unpublished	State Anxiety *p* = 0.864 *d* = −0.16 Trait Anxiety *p* = 0.350 *d* = −0.12	Some concerns	- Unnecessary as others small also. -Assessors were not blind -Baseline scores below the cut-off for clinical anxiety and healthy population. -Experimenter administered Reiki. -Baseline differences not statistically analyzed.

**Clinical stress GRADE: high**			
Bowden et al. (2011) Mood and wellbeing in High vs. Low Anxiety and Depression.	**Sig** **High Stress Reiki > placebo (*p* = 0.008). *d* = 0.87 *d* = 0.90 at f.up.** Not sig in low mood (*p* < 0.05) *d*	Some concerns	-Baseline scores for wtress higher in Reiki group. -Author administered Reiki but was unlikely to bias outcome as sat behind blindfolded SS with no touch (blinding successful *p* < 0.05) -No pre-specified plan.

Vasudev and Shastri (2016) Self-perceived work-related stress software professionals.	**Hands on Reiki and DR Placebo *p* = 0.028 *d* = 0.63** No diff bet hands on Reiki and DR *p* = 0.878 suggests benefits from hands on R not due to touch or other placebo effects.	Some concerns	-Hands on and distant Reiki was delivered by the experimenter but instruction was given which should have reduced placebo bias. -Hands on Reiki > DR placebo and no difference bet hands on Reiki and DR suggesting Reiki is not influenced by the placebo factors. -31% dropout rate (raw scores in thesis) though reasons mostly random.

Yüce and Taşcı (2021) Stressed Caregivers of cancer patients.	**CSI *p* < 0.001 at post-treatment 6 weeks. *d* = 2.3**	Some concerns	-Investigator administered Reiki and trained the sham group. -17% dropouts and no ITT analysis were reported.

**Stress: normal range Grade: low to moderate.**			
Bowden et al. (2010) Well-being and salivary cortisol in healthy psych undergraduates.	NS (*p* = 0.054) stress subscale over placebo on DASS.	Some concerns	-Higher pre-stress scores in the Reiki group may have assisted Reiki -Author administered Reiki but was unlikely to influence as sat behind a blindfolded SS with no touch (blinding successful *p* < 0.05) -No pre-specified plan and 14% dropouts.

Shore (2004) Depression and Self Perceived Stress.	**Perceived Stress Scale (PSS) post-test *p* = 0.029 *d* = 0.88** **1 year follow up *p* = 0.001 *d* = 2.02**	Some concerns	-High drop out from post-treatment to f. up through analysis of original numbers (73 to 46). -Placebo R believed Hands On R was the placebo to reduce expectancy. - PSS-10 may not have captured stress adequately.

**Clinical depression GRADE: high**			
Dressen and Singg (1998) Chronically ill and in pain.	**Reiki sig > placebo (no *p*-value)** ***P* = 0.0001 pre-post. *d* = 1.4 > placebo**	Some concerns	-Unstated if assessors were blind. -Subjects were self-selected in response to an advertisement. -No pre-specified analysis plan was found. -Follow up Reiki scores were not compared to other groups

Erdogan and Cinar (2016) Depression in the Elderly.	**Reiki sig > placebo at all 4 time measurements.** **1st *p* = 0.001 4th *p* = 0.000** **8th *p* = 0.000, 12th *p* = 0.000 (1 mthf.up). Inadequate data to calculate *d*.**	Some concerns	-One of only 2 studies blinding sham practitioners, i.e., Reiki done by the researcher but Placebo Reiki practitioners believed they were doing Reiki to control for practitioner expectancy effects. -NI whether outcome assessors were blind.

Shore (2004) Depression and Self Perceived Stress.	**Reiki > placebo Reiki *p* = 0.042 *d* = 0.74** **1 yr follow up *p* = 0.001 *d* = 1.43**	Some concerns	-High drop out from post-treatment to f. up through analysis on original numbers (73 to 46) -Placebo R led to believe Hands On was the placebo grp to reduce expectancy. -SS self-selected in response to an advertisement as being in need of treatment for self-perceived depression and stress/anxiety.

Shiflett et al. (2002) Functional Recovery Post Stroke Rehab.	NS Depression (*p* > 0.05). Inadequate data to calculate *d*-value.	HIGH	-double-blinded, i.e., blinded practitioners and participants. -blinding practitioners were successful but attunement may have not been successful. -20 historic controls used. -16% missing data and cognitive FIM missing. ITT not done. -No pre-specified analysis plan. -Variation of 6–10 treatments/grp and though not related to group assignment may have still influenced outcomes.

**Depression: normal range** **GRADE: low**			
Bowden et al. (2010) Well-being and salivary cortisol in healthy psych undergraduates.	NS (*p* > 0.05 no values given).	Some concerns	-Author administered Reiki but was unlikely to influence as sat behind a blindfolded SS with no touch (blinding successful *p* < 0.05) -No pre-specified plan. -Dropouts 14%

Bowden et al. (2011) Mood and wellbeing in High vs. Low Anxiety and Depression.	NS (*p* > 0.05 no values given)	Some concerns	-Experimenter administered Reiki. -Participant blinding was assessed as successful. -Baseline scores for stress were higher in the Reiki group.

**Burnout** **GRADE: low to moderate**			
Díaz-Rodríguez et al. (2011a) Nurses with Burnout.	**Reiki reduced diastolic BP > placebo *p* = 0.04 *d* = 0.59 and increased SIgA over placebo *p* = 0.04 *d* = −0.75** Systolic BP *p* = 0.24 a-amylase activity *p* = 0.71	Some concerns	NI found on pre-register so maybe some concerns are there. Some research questions the use of biological markers to measure burnout and some supports it.

Díaz-Rodríguez et al. (2011b) Health Care Proff with Burnout. –	**ECG recordings for SDNN Reiki > placebo (*p* < 0.04), *d* = 0.71** **Body temp Reiki > Placebo (*p* = 0.02), *d* = 0.85** Salivary Cortisol *p* = 0.08, ECG RMSSD *p* = 0.06 HRV non-sig.	Some concerns	NI found on pre-register so maybe some concerns. -blinding of participants was tested and found to be successful. - Greater significance across other measures may have resulted from longer treatments and multiple sessions as NS was borderline. Some research questions the use of biological markers to measure burnout and some supports it.

Rosada et al. (2015) Burnout Health care professional.	**Reiki > placebo Overall for burnout *p* = 0.011.** **Reiki also reduced emotional exhaustion, depersonalization, and increased pers accomplishment (*p* < 0.05 no values given)**. **Inadequate data to calculate *d*.**	Some concerns	-Unstated whether outcome assessors were blind. -Published study excludes some non-significant results from the original thesis. -baseline numbers between groups for single people were not given and this is important to some results.

*Bold indicates statistically significant results and moderate or larger effect sizes. NI means no information.*

Attempts were made to contact the authors of all the 14 studies to clarify unclear or missing information and provide a fair review. Author contacts were searched for through Google, LinkedIn, and Research Gate. No contact details were found for the authors of one RPCT (Shiflett et al., 2002). Of the remaining 13 RPCTs, the authors of four studies responded (Thornton, 1991; Vasudev and Shastri, 2016; Baldwin et al., 2017; Çinar et al., 2022).

## Results

The search found 26 randomized placebo controlled trials (RPCTs) that examined *hands-on* Reiki’s effectiveness over placebo in adults using valid outcome measures translated in English (Thornton, 1991; Dressen and Singg, 1998; Witte and Dundes, 2001; Shiflett et al., 2002; Mackay et al., 2004; Shore, 2004; Gillespie et al., 2007; Assefi et al., 2008; Bowden et al., 2010, 2011; Catlin and Taylor-Ford, 2011; Díaz-Rodríguez et al., 2011a,b; Ventura Carraca, 2012; Baldwin et al., 2013, 2017; Fortes Salles et al., 2014; Novoa and Cain, 2014; Rosada et al., 2015; Alarcao and Fonseca, 2016; Erdogan and Cinar, 2016; Midilli and Gunduzoglu, 2016; Vasudev and Shastri, 2016; Bat, 2021; Yüce and Taşcı, 2021; Çinar et al., 2022). Fourteen of these met PICO for examining the effectiveness of Reiki over placebo in measuring symptoms of mental health (Thornton, 1991; Dressen and Singg, 1998; Shiflett et al., 2002; Shore, 2004; Bowden et al., 2010, 2011; Díaz-Rodríguez et al., 2011a,b; Rosada et al., 2015; Erdogan and Cinar, 2016; Vasudev and Shastri, 2016; Baldwin et al., 2017; Yüce and Taşcı, 2021; Çinar et al., 2022). These RPCTs included outcome measures of depression, anxiety, stress, and burnout. No studies in this SR reported adverse effects of Reiki.

### Anxiety and Stress

Eight peer-reviewed and one non-peer-reviewed RPCTs using standardized outcome measures for anxiety and stress met the criteria for inclusion. Six included measures for anxiety (Thornton, 1991; Dressen and Singg, 1998; Bowden et al., 2010, 2011; Baldwin et al., 2017; Çinar et al., 2022), and five included measures for stress (Shore, 2004; Bowden et al., 2010, 2011; Vasudev and Shastri, 2016; Yüce and Taşcı, 2021).

Thornton (1991) conducted an unpublished RPCT on the effect of Reiki on adult healthy female nursing students (Thornton, 1991). In this study, 42 healthy student nurses were randomly assigned to either 1 h of Reiki (*n* = 22) or 1 h of mock Reiki from a research assistant (*n* = 20). The Spielberger State-Trait Anxiety Inventory was given pre-post treatment. State and trait anxiety were lower in both placebo and Reiki groups post-treatment; however, there were no between-group differences between Reiki and sham groups for state anxiety (*p* = 0.864) or trait anxiety (*p* = 0.35) post-treatment. By direct contact with the author, participants were randomized using a random numbers table. It is unclear if the groups were equal at baseline as differences were not statistically analyzed. All mean baseline scores (ranging from 34 to 36) were under the cutoff of 39 for both state and trait anxiety. These scores are considered clinically insignificant (Julian, 2011) and likely decreased the impact of Reiki in this study. The small sample size may have also reduced power.

Contact with the author revealed that the assessors were not blind and that Reiki was delivered by the author, a Reiki master with 10 years of experience. Mainly the unblind assessors and author delivery of Reiki leave this study with some concerns for ROB. This study does not support the effectiveness of Reiki over placebo in populations with anxiety in the normal range.

A larger study on adults who had been chronically ill for at least 1 year with chronic pain was conducted by Dressen and Singg (1998). One hundred and twenty participants were randomly assigned to one of four groups: (1) Reiki, (2) progressive muscle relaxation, (3) mock Reiki, and (4) the control group that received no treatment but came to read any material of their choice. Sessions lasted 30 min and were conducted two times a week for 5 weeks. Forty-five percent of the participants experienced pain from chronic headaches. Other categories included pain from cancer, coronary heart disease, arthritis, and hypertension. A sizeable significant reduction in pain intensity global (*p* = 0.0001), depression (*p* = 0.0001), state anxiety (*p* = 0.0001), and trait anxiety (*p* = 0.0001) was found in the Reiki groups post-treatment, and it was stated that Reiki was significantly more effective than placebo in all the areas (between-group *p*-values not given). Large effect sizes were found for Reiki’s effect over placebo for reducing state anxiety (*d* = 1.36), trait anxiety (*d* = 1.07), and depression (*d* = 1.4). Also relevant to mental health, Reiki was stated to be significantly more effective than placebo in improving self-esteem, loss of control, and an unrealistic sense of control as measured with validated outcome measures (*p*-values not given). The subjects were partly self-selected, and it was unstated whether the assessors were blind to treatment assignment. No prespecified analysis plan was found, and a 3-month follow-up was only assessed for the Reiki group. Despite some concerns regarding ROB, this study supports the effectiveness of Reiki in treating anxiety over placebo in those with chronic pain when the treatment is provided two times a week for 5 weeks.

Bowden et al. (2010, 2011) conducted two studies that included measures for anxiety, stress, and depression. In the first study, Bowden et al. (2010) examined the effect of Reiki and positive imagery on the wellbeing of and salivary cortisol in 35 healthy psychology undergraduates with mental health scores all in the normal range. The participants were randomly assigned to one of the six groups, and all the groups were asked to engage in a self-hypnosis/relaxation exercise. Three groups (*n* = 18) underwent Reiki 3–30 inches above their head with the experimenter seated behind them. The other three groups were not administered Reiki but were told that they were receiving Reiki (*n* = 17) while the experimenter sat behind them with their hands at their side. Ten 20-min sessions were given to both groups from between 2 and 12.5 weeks. All the groups were blindfolded or wore headphones depending on the task. Although the experimenter conducted Reiki in the study, the additional steps to blind the subjects should have reduced the influence of placebo. Blinding was tested and found to be successful. Pre- and post-assessments were conducted by a co-experimenter blind to the treatment groups. In the first study, despite within-group improvements in anxiety (*p* = 0.037) and stress (*p* = 0.001) but not in depression (*p* = 0.102), there were no significant between-group differences in the results for Reiki over placebo for anxiety (*p* = 0.295), stress (*p* = 0.057), or depression (*p* = 0.152). Treatment delivery ranged from 2 to 12.5 weeks; however, statistical analysis found this to not to have an impact on outcomes. No prespecified analysis plan was found, and “wellbeing” was operationalized with the Depression, Anxiety and Stress Scale (DASS). This is not a measure of wellbeing and likely not a sensitive measure for participants all scoring in the normal range at baseline. There was also a 14% dropout rate with no ITT analysis. Overall, this study presents an ROB of some concern. This study does not support the effectiveness of Reiki over placebo for participants with mental health outcome scores in the normal range.

In a replication of the above study (Bowden et al., 2011), the same authors examined whether Reiki benefited mood and wellbeing, but this time, they controlled for mood. Forty university students, half with high anxiety and/or depression and half with low anxiety and/or depression, were randomly assigned to Reiki or placebo Reiki groups in the same arrangement as the previous study. Despite being grouped separately according to “high” and “low” levels of anxiety/depression, baseline scores for the high mood groups still nearly all fell in the normal range on the DASS. Depression baseline means were “normal,” and anxiety means were borderline normal at a cutoff of 7 and “high” group stress scores were in the clinical range (Lovibond and Lovibond, 1995). On the HADS, scores were in the mild to moderate range, but no separate baseline scores were provided for anxiety and depression, so this measure could not be assessed in this review.

Six 1/2 hour treatment sessions were administered between 2 and 8 weeks. Again, the impact of this variation was statistically tested and found to not impact the outcomes. Blinding was also tested and found to be successful. It is unstated whether the assessors were blind, but it was expected given that it is a replication of their previous study, which had blinded assessors (Bowden et al., 2010) and the care taken to blind the participants. There was no ITT analysis; however, there was a low dropout rate of 7%.

Reiki reduced total DASS scores more than placebo for the high mood group only, and although this did not reach significance post-treatment (*p* = 0.075), it was significant at follow-up (*p* = 0.045). Reiki significantly reduced stress compared with placebo in the high mood group post-treatment (*p* = 0.008) but did not reduce anxiety compared with placebo in high mood (*p* = 0.084) or depression (no between-group means given). Effect sizes for Reiki compared with placebo for stress were also large in the high mood group post-treatment (*d* = 0.87) and at follow-up (*d* = 0.9).

There was no prespecified analysis plan found for this study. It is likely that the low sample size of ten per subgroup and low baseline scores reduced the outcomes of significance. Overall, this study presents an ROB of some concerns. These results provide support for Reiki’s influence over placebo at reducing clinical levels of stress but not at reducing anxiety or depression in the normal range.

Baldwin et al. (2017) examined the effects of Reiki on pain, anxiety, and blood pressure in patients undergoing knee replacement surgery. In this study, 46 adults scheduled for single knee replacement surgery were randomly assigned to one of three groups, all incorporating standard hospital care (Baldwin et al., 2017): (1) three or four 30-min Reiki treatments by a Reiki master, (2) three or four 30-min sham Reiki sessions with an untrained person following the same hand positions as the Reiki master from a printed protocol, and (3) three or four sessions of quiet time. The participants and assessors were blind to treatment assignment. Blinding was assessed and found to be successful overall. The first session was 1 h prior to surgery, and subsequent sessions 24, 48, and 72 h after the surgery if not already discharged. Data were deidentified and collected by trained data collectors. Anxiety was measured with the State-Trait Anxiety Inventory, and the means met cutoffs for clinically relevant anxiety at baseline (Julian, 2011).

Only the Reiki group showed significantly reduced state anxiety scores as measured on the State-Trait Anxiety scale at discharge (*p* = 0.004). The placebo group was non-significant; however, the Reiki group could not be directly compared to the placebo group, because the placebo group was too small for valid statistical comparison because of dropouts. The magnitude of effect for Reiki over placebo on anxiety was large (*d* = 0.93). Despite no ITT analysis being performed, post-baseline dropouts were controlled for by statistical analysis. Data from 48-h post-surgery were treated separately by paired *t*-tests, which compared pre-intervention and pre-surgery data, with patients discharged 48 h or more post-surgery.

If the Reiki sample size better matched that of the smaller placebo group, Reiki may not have performed as well as it did. No prespecified analysis plan was found. Overall, this study presents an ROB of some concerns, and the outcomes suggest that Reiki was more effective than placebo in reducing state anxiety in populations with clinically relevant anxiety levels.

A recent study examined the effect of Reiki on stress levels of caregivers of patients with cancer (Yüce and Taşcı, 2021). In this study, 42 caregivers were randomly allocated to 45 min of Reiki (*n* = 21) or sham Reiki (*n* = 21) once a week for 6 weeks. The caregivers met the cutoff scores for high stress on the standardized Caregiver Strain Index (CSI). They and the assessors were blind to treatment allocation. The investigator delivered Reiki and trained four nursing students to identically deliver the sham Reiki, increasing ROB. Though a strict application protocol was followed. All baseline measures were similar between the groups. Reiki performed significantly better than placebo with high statistical significance on all measures except for salivary cortisol. On week 6, the Reiki group had significantly lower stress (CSI) scores than the sham Reiki group (*p* < 0.001) with a very large magnitude of effect (*d* = 2.3). A prespecified plan was reported in clinicaltrials.gov. There was also a 17% dropout rate with no evidence of an ITT analysis. Because of these issues, this study had an ROB of some concerns. However, it provides support for Reiki over placebo in treating high levels of caregiver stress.

Another recent study evaluated the impact of Reiki on pain, wellbeing, and anxiety in Turkish hospital patients treated for pain from fibromyalgia (Çinar et al., 2022). Fifty patients were randomly and blindly assigned to either 30 min of Reiki or sham Reiki one time a week for 4 weeks, with 25 patients in each group. The assessors were blind to the treatment group. Only after the fourth treatment was Reiki found to reduce both state anxiety (*p* = 0.005) and trait anxiety (*p* = 0.003) significantly more than placebo. The data required to calculate effect sizes could not be obtained from the author and therefore could not be included. There were no baseline differences and no dropouts. No prespecified analysis plan was found for this study. Overall, it was assessed as having an ROB of some concerns but provided evidence that Reiki can reduce state and trait anxiety compared with placebo in patients who have fibromyalgia after four 30-min sessions.

### Self-Perceived Stress

Two peer-reviewed RPCTs have focused on the effects of Reiki on self-perceived stress. Shore (2004) examined the long-term effects of Reiki on depression and self-perceived stress. Forty-six adults were randomly selected from a pool of respondents to an advertisement for those seeking treatment for symptoms of depression and stress. The participants were randomly assigned to hands-on Reiki, distant Reiki, or distant Reiki placebo and were blind to their treatment group. Reiki was performed by an independent healer. The assessors were blind to group assignment. All the groups (except placebo) were given 1 to 1.5 h of Reiki one time a week for 6 weeks. Those in the distant Reiki condition were in an identical room to those receiving hands-on Reiki and were given the treatment from afar. Those in the placebo distant Reiki condition were also in an identical room and were told that they were given the treatment from afar but were not given any treatment. The author states on page 43 that, “Participants in the hands-on Reiki condition believed they were receiving mock Reiki and participants in the placebo distant Reiki condition believed they were receiving distant Reiki (Shore, 2004).” This was conducted to reduce the effect of placebo further but would have biased the outcome in favor of placebo in how the groups were treated unequally. Despite this, the study still produced results with high significance in favour of Reiki.

Baseline means were clinically relevant in the mild depression range on the BDI. The means for the perceived stress scale were very low at baseline and in the normal range. Post-treatment showed a significant reduction in symptoms between the hands-on Reiki and distant placebo Reiki treatment groups on the Perceived Stress Scale (PSS) at post-test (*p* = 0.029) and at 1-year follow-up (*p* = 0.001). Effect sizes for perceived stress were also large at post-treatment (*d* = 0.88) and very large at follow-up (*d* = 1.43). There was also a significant reduction in depression scores on the Beck Depression Inventory (BDI) at post-treatment (*p* = 0.042) and at 1-year follow-up (*p* = 0.001), with corresponding effect sizes of moderate at post-treatment (*d* = 0.74) to very large at follow-up (*d* = 1.43). Significant outcomes were also found for Reiki over placebo on the Beck Hopelessness Scale (BHS) at post-treatment (*p* = 0.019) and 1-year follow-up (*p* = 0.009). Large effects of magnitude and significance at follow-up should be treated with caution because of very high dropouts (40%) and no ITT analysis. However, the reasons participants dropped out appeared to be random, reducing bias from attrition. Initially, this study was published as a dissertation.

This study shows an ROB of some concerns mainly because of high follow-up attrition. It provides support for Reiki in reducing normal levels of stress and mild clinical depression over placebo in the short term and possibly with long-term effects.

Vasudev and Shastri (2016) studied 120 software professionals suffering mainly from work-related stress and working at a firm in Bangalore, India. The participants were randomly assigned to hands-on Reiki, distant Reiki, distant placebo Reiki, or no Reiki (control). Reiki was applied in the treatment groups 5 min per day for 21 days. The placebo participants were told that they would receive Reiki, but they did not, to control for expectancy effects, again biasing the outcomes in favor of placebo. All the four groups were assessed pre- and post-21 days of treatment with the 14-item Perceived Stress Scale. Direct contact with the author confirmed that the assessor was blind to group assignment. In the original thesis, additional scales were also used as follows: Coping Checklist (Rao et al.) and WHO (Five) wellbeing index, sociodemographic checklist, and stressor inventory (Vasudev and Shastri, 2016).

In this study, a significant difference between groups occurred for perceived stress, but it did not specify which of the three groups were compared. However, the original thesis identified that a significant difference between hands-on Reiki and distant placebo Reiki (*p* = 0.028) was found. This had a medium magnitude of effect (*d* = 0.63). The experimenter administering Reiki used hands-on elements such as touch and body language which could have increased the ROB favoring the hands-on Reiki treatment condition. However, there was no statistical difference between the hands-on Reiki and distant Reiki treatment conditions (*p* = 0.878), and distant Reiki was also more effective than placebo distant Reiki (*p* = 0.019). This suggests that placebo factors of experimenter bias and the hands-on placebo elements of touch and body language were not significant.

The thesis identified several other omissions from the published article that favored both experimental and placebo outcomes and were therefore unlikely due to bias. Direct feedback from the author supports this: “The publisher said I had too much content, so I had to cut it short.” There was a high dropout rate of 31%. No ITT analysis was confirmed by direct contact with the author. The author stated the main reason for attrition was being unable to maintain regular attendance at the sessions, so it is unclear if this was random. Although Reiki was given with high frequency (21 days), it was only given for 5 min at a time. A greater effect may have been found with a longer treatment period. This study showed an ROB of some concerns mainly because of high attrition rates and no ITT analysis but supports the use of Reiki over placebo for normal levels of stress.

### Conclusion on the Effects of Reiki on Anxiety

All of the above six studies measuring the effects of Reiki on anxiety showed an ROB of some concern, but none had methodological issues too great to suggest that the outcomes were significantly compromised. Of the three RPCTs measuring clinically relevant anxiety, two had significantly reduced anxiety in the Reiki group compared with placebo (Dressen and Singg, 1998; Çinar et al., 2022). The other (Baldwin et al., 2017) did not directly compare the Reiki and placebo groups, but only the Reiki group had significantly reduced anxiety. Where effects could be calculated in two studies, large to very large magnitudes of effect for Reiki over placebo were found for treating anxiety. This provides support that Reiki can reduce anxiety compared with placebo in people with clinically relevant levels of anxiety.

The three RPCTs that did not meet clinical cutoffs for anxiety produced non-significant results directly comparing placebo and Reiki for anxiety (Thornton, 1991; Bowden et al., 2010, 2011). This suggests that Reiki is not more effective than placebo in reducing anxiety in the normal range.

### Conclusion on Reiki’s Impact on Stress

None of the above five studies measuring the effects of Reiki on stress showed a ROB, which would suggest that the outcomes were seriously compromised, that is, all ROBs were of some concern (Shore, 2004; Bowden et al., 2010, 2011; Vasudev and Shastri, 2016; Yüce and Taşcı, 2021). For clinically relevant outcomes, Reiki was found to be highly significant compared with placebo, and with medium to very large effect sizes in three studies (Bowden et al., 2011; Vasudev and Shastri, 2016; Yüce and Taşcı, 2021). Reiki was also suggested to be effective with those experiencing normal levels of stress. Reiki showed significant effects over placebo, with a large magnitude of effect on reducing stress in Shore (2004) et al.’s (2010) study. These results suggest that, compared with placebo, Reiki assists in reducing clinically relevant stress and may reduce normal levels of stress.

### Depressed Mood

There have been six RCPTs in which Reiki has been compared to placebo with reducing scores on validated outcome measures of depression (Dressen and Singg, 1998; Shiflett et al., 2002; Shore, 2004; Bowden et al., 2010, 2011; Erdogan and Cinar, 2016).

Reiki significantly reduced the symptoms of depression in chronically ill patients (Dressen and Singg, 1998). This study has previously been reviewed in the section on anxiety. The study’s largest significant treatment effect was found for Reiki on treating depression as measured by the Beck Depression Inventory 11 (*p* = 0.0001 post-treatment). Reiki was also significantly more effective than placebo (no between group *p*-value was provided), and the magnitude of the effect was large (*d* = 1.4). Depression baseline means were all clinically relevant and in the mild range for women and moderate range for men. This study was assessed as having an ROB of some concerns and supports the hypothesis that Reiki reduces clinical levels of depression compared with placebo in the short-term.

A study on functional recovery for patients undergoing post-stroke rehabilitation (Shiflett et al., 2002) included outcome measures for the effect of Reiki on depression. Here, 30 patients were randomly allocated to Reiki by a Reiki master (*n* = 10), a Reiki novice (*n* = 10), or a sham Reiki (*n* = 10). An additional twenty historic control patients were used. Six to ten 30-min sessions were given over 2.5 weeks for each of the Reiki, Reiki novice, and sham Reiki conditions. This was double-blinded in that the Reiki novice and sham Reiki practitioners did not know whether they had been attuned with Reiki. Various analyses suggest that this was likely successful. The outcome measure for depression was The Centre for Epidemiological Studies Depression Scale (CES-D). The CES-D baseline cutoff means for clinical levels of depression were low but mostly met. This study did not support the use of Reiki for clinical levels of depression. However, there were significant methodological problems. The baseline scores revealed statistically significant differences between age and severity of impairment. These were used as covariates to correct for this difference, but this still may have influenced outcomes. The method of Reiki attunement was questionable, as it appeared to have been conducted at a distance (not hands-on) with all the practitioners together in the same room. The arrangement for blinding the Reiki practitioners meant that the novice practitioners used in the study had no experience with a basic level of training having been attuned to level 1. Reiki is believed to flow more strongly with higher levels of attunement and more years of experience, so this is not ideal. There were an uneven number of treatments of 6 to 10 for each condition, although it stated that this was unrelated to group assignment, functional status, mood, or FIM score. The dropout rates were at least 16%, and no ITT analysis was conducted. It is not stated whether the outcome assessors were blind, although we might expect so given that the therapists and participants were blind. The cognitive portion of the FIM was missing, which likely influenced the outcomes for functional recovery and raised issues regarding general data storage. There was also no prespecified analysis plan. Overall, this study’s ROB was assessed as high, and the results should be treated with caution. Also, effect sizes could not be calculated because of insufficient data.

Shore (2004) examined the long-term effects of Reiki on depression and self-perceived stress in volunteers. This study was reviewed in the section on stress. BDI scores were above the cutoff for being mildly clinically depressed. Reiki resulted in a significant reduction in depression compared with placebo on the Beck Depression Inventory (*p* = 0.042) and maintained this at 1-year follow up (*p* = 0.001), along with medium effects at post-treatment (*d* = 0.74) to very large effects at follow-up (*d* = 1.43). The ROB for this study was assessed as having some concerns, but it provided evidence that Reiki reduces depression compared with placebo in the short term and possibly in the long term in people who are mildly clinically depressed.

Two RPCTs by Bowden et al. (2010, 2011), where the subjects exhibited normal baseline scores for depression, showed no evidence that Reiki had a therapeutic effect when compared with placebo Reiki (Bowden et al., 2010, 2011). These studies have been reviewed in the sections on anxiety and stress, and both were assessed as having an ROB of some concerns.

In another RPCT, 90 elderly depressed volunteers living in a nursing home in Istanbul (Erdogan and Cinar, 2016) were randomly and blindly allocated to 45- to 60-min sessions one time a week for 8 weeks. The sessions were Reiki, sham Reiki, or a waitlist control. The therapist was “a researcher who (was) a Reiki master (p37),” and it was unclear if this was the author of the study. The sham Reiki group was applied by four nurses who did not have training in Reiki but believed that they were practicing Reiki. This should have helped to control for differences in expectancy effects between the Reiki and placebo Reiki practitioners. In this way, this study controlled for both participant and practitioner biases. There appear to be no dropouts in this study. Reiki was applied in the same room and at the same time as real Reiki. The outcomes were measured with the Geriatric Depression Scale (GDS) after the 1st, 4th, 8th, and 12th weeks (i.e., at 1-month follow-up), and Reiki was found to be significantly more effective at reducing depression than both placebo Reiki and the control across all four-time measurements over the 12 weeks with highly statistically significant outcomes at every measure (*p* = 0.001 post week 1 and *p* = 0 thereafter). Effect sizes could not be calculated because of insufficient data. All the participants scored at least 14 on the GDS at baseline, falling at least in the moderately clinically depressed range (Laudisio et al., 2018). It is not clear how the 45- to 60-min treatment time was distributed between conditions, but this was unlikely to account for such high levels of difference between groups. It is also not stated whether the researcher, who was the outcome assessor, was blind to the treatment assignment. Overall, this study was assessed as having some concerns but provides evidence that Reiki is more effective than placebo in reducing depression in the short term and the long term in moderately depressed elderly populations.

### Conclusion on Reiki’s Impact on Depression

Of the six Reiki RPCTs measuring depression, only one RPCT had significant methodological issues that may have resulted in invalid outcomes (Shiflett et al., 2002). The remaining five studies all showed an ROB of some concerns. For the two RPCTs in which baseline scores for depression were in the normal range, Reiki had no effects of significance over placebo (Bowden et al., 2010, 2011). In the other three RPCT where baseline scores were clinically relevant, all found that Reiki was more effective than placebo at reducing depression (Dressen and Singg, 1998; Shore, 2004; Erdogan and Cinar, 2016), and where calculations were possible, in studies with clinically relevant baselines, all showed large to very large effect sizes. This provides support that Reiki is effective in reducing clinically relevant symptoms of depression compared with placebo but does not support Reiki’s ability to reduce depression in the normal range.

### Burnout

Three RCPTs have examined the effects of Reiki on burnout over placebo (Díaz-Rodríguez et al., 2011a,b; Rosada et al., 2015), two of which used biomarkers to measure burnout (Díaz-Rodríguez et al., 2011a,b). In these studies, the author cited literature which states that the stress response stimulates the sympathetic nervous system and blood pressure and that several biological markers are often used to measure stress. While some literature supports the relationship between stress and blood pressure (Gasperin et al., 2009; Ayada et al., 2015) and the relationship between biomarkers and burnout (Deneva et al., 2019; Bayes et al., 2021), some research does not (Danhof-Pont et al., 2011).

Díaz-Rodríguez et al. (2011a) examined whether Reiki given to nurses with burnout syndrome had beneficial effects on biomarkers for burnout and stress. The biomarkers measured were concentrations of salivary IgA, α-amylase activity, and blood pressure. Eighteen adult nurses diagnosed with burnout syndrome by a psychologist using the Maslach Burnout inventory Manual were randomly assigned to 30 min of Reiki or sham Reiki. The participants and data collectors were blind. Blood pressure was measured with an Omron HEM-737 validated device, with measurements performed in triplicate and the average taken for analysis. There were no dropouts. The Reiki group produced a significant decrease in diastolic blood pressure compared to placebo (*p* = 0.04) with a medium magnitude of effect (*d* = 0.59). Reiki also showed a significant increase in SigA concentration compared to placebo (*p* = 0.04) with a medium magnitude of effect (*d* = –0.75). The magnitude is negative because of an increase in SigA, which suggests an improvement in immune function. Compared with placebo, Reiki had no significant effect on systolic blood pressure (*p* = 0.24) or a-amylase activity (*p* = 0.71). It was concluded that a single 30-min session of Reiki led to immediate improvement in the innate immune function (SigA) and blood pressure regulation, which the placebo effect could not explain. A prespecified analysis plan was not found. This study was assessed to have some concerns. It provides support for Reiki in treating biomarkers of burnout.

In a similar study (Díaz-Rodríguez et al., 2011b), 21 healthcare professionals diagnosed with burnout by a psychologist using criteria from the Maslach Burnout Inventory Manual were randomly assigned to 30 min of Reiki or sham Reiki. Again, biomarkers of burnout and stress were measured using validated instruments and standardized procedures. The biomarkers measured were heart rate variability (HRV), cortisol from salivary flow rate, and body temperature (which used the OMRON Gentle Temp 510). The participants were randomly assigned, and the participants and data collectors were blind. There were no dropouts. Only 19% of the participants identified their treatment group correctly, so blinding was successful. All the sessions occurred in the morning between 9 am and 12 pm, and the subjects abstained from food, alcohol, caffeine, and exercise 2 h prior to assessments. Interventions were given after 20 min of rest. ECG recordings for SDNN were significantly higher than placebo (*p* < 0.04.) with a moderate effect (*d* = 0.71). Body temperature was significantly higher than placebo after Reiki (*p* = 0.02) with a large magnitude of effect (*d* = 0.85). The authors stated that the higher body temperature was significantly correlated with the LF domain after Reiki (*p* = 0.02), suggesting a therapeutic effect on the parasympathetic nervous system. When compared with placebo, Reiki had no significant effect on salivary cortisol (*p* = 0.08) and ECG RMSSD (*p* = 0.06). The authors concluded that Reiki positively influenced the parasympathetic nervous system when applied to the healthcare professionals with burnout syndrome. Once again, a prespecified analysis plan was not found. This study was assessed to having an ROB of some concerns. It supports the use of Reiki for treating biomarkers of burnout.

Reiki was also found to reduce burnout among community mental health clinicians (Rosada et al., 2015). Forty-five mental health clinicians were randomly and blindly allocated to 30 min of Reiki one time a week for 6 weeks or 30 min of sham Reiki one time a week for 6 weeks in a randomized controlled crossover design. Burnout was measured with the Maslach Burnout Inventory. The participants were self-selected volunteers working at mental health agencies. There was no information on whether the assessors were blind to the treatment assignment, and the dropout rate was 4%. Reiki was more effective than placebo Reiki overall in decreasing burnout (*p* = 0.011). When compared with placebo, Reiki also significantly reduced burnout, as evidenced by decreased emotional exhaustion, decreased depersonalization, and increased personal accomplishment on the scale (*p-*values not provided). Effect sizes could not be calculated because of insufficient data. The 6-week washout period in this crossover design appears to have prevented a carryover effect because when the Reiki treatment was provided in the first 6 weeks, it was more effective than when it was provided in the second 6 weeks. The original more detailed thesis describes two additional assessments: the Social Readjustment Rating Scale (SRRS) and the Perceived Self Efficacy Scale. These both produced insignificant results. Furthermore, although overall Reiki was significantly more effective than sham Reiki on the Maslach Burnout Inventory (MBI) (*p* = 0.011), it was only significant on the individual scales of the MBI and on the primary symptom of the MYMOP for single people (*p* not provided). While being single may be relevant to burnout, the proportion of single people was not reported in baseline characteristics, which are important to these outcomes. This selective reporting and the absence of information about the blinding of assessors leave this study with an ROB of some concerns.

### Conclusion on Reiki’s Impact on Burnout

Of the three RPCTs conducted on the influence of Reiki over placebo to reduce burnout, none had significant methodological issues, which would suggest the outcomes to be invalid. All the three RPCTs found significant effects for Reiki over placebo in treating burnout in healthcare professionals with mostly moderate effect sizes for those that could be calculated. The three studies provide evidence that Reiki reduces burnout over placebo in healthcare professionals in the short term.

## Grade Ranking

In accordance with GRADE guidelines, a GRADE ranking was applied to each mental health area under study (Brozek et al., 2021; Schünemann, 2022; Siemieniuk, 2022). It is often reported that Reiki energy flows according to the degree of imbalance a person is experiencing and when it is needed (Webster, 2016; Powers, 2018; Frazier, 2020) and stops flowing when the balance is restored. Given this, it was expected that the more clinically unwell the population under study, the more therapeutic impact the Reiki would have. A number of studies in this SR used healthy populations with mental health scores in the normal range, in other words, scores that were clinically irrelevant. It was expected that Reiki would have a minimal effect over placebo in these populations, just as one might expect almost any treatment to produce a limited effect on a healthy normal individual with nothing to treat. This appeared to be the case. Most studies did not screen for a diagnosis of mental health, particularly, as many used healthy participants.

To encourage consistency in the populations of studies under review, standardized mental health outcome measurements were grouped into populations with baseline means reaching cutoffs for clinical levels of mental health (low range or higher) and studies with baseline means falling in the normal range. Burnout was not grouped this way because, in two studies, the population met a diagnosis of burnout, and in the other study, it was not assessed. In this study, health professionals were self-selected for burnout, and it was not stated whether cutoff scores for burnout were met (Rosada et al., 2015). Means were also not provided.

GRADE is assessed according to the following criteria: ROB, inconsistency, indirectness, imprecision, and publication bias. The studies are then checked for factors that may upgrade the GRADE assessment (Schünemann, 2022; Siemieniuk, 2022). According to GRADE guidelines an RCT, and more so one that controls for placebo, begins at a GRADE ranking of *High*. In the ROB criteria, only one study showed high ROB (Shiflett et al., 2002), and this showed non-significant results for those with clinically relevant scores of low depression. All the other studies showed an ROB of some concerns, but none had methodological issues serious enough to suggest that the results were invalid. It is important to note that an ROB of some concerns does not necessarily affect GRADE outcomes, as the ranking is not a result of averaging the ROB across studies but carefully considering the contribution of each study, and they recommend this to be done conservatively (Schünemann et al., 2013). “One should be conservative in the judgment of rating down. That is, one should be confident that there is substantial risk of bias across most of the body of available evidence before one rates down for risk of bias (5.2.1).”

With respect to the criteria of inconsistency, this was assessed as follows: populations were drawn from university hospitals (4 studies), undergraduate students (3 studies), self-selected or GP referred (3), one acute care hospital, one community mental health agency, one rehabilitation institute, and one nursing home. Six studies were conducted in the United States, three in Turkey, two in the United Kingdom, two in Spain, and one in India. One study used 58% men, three gave no percentages, another three used 100% women, and the remainder used over 50% women.

Despite this variation in settings, country, and gender, no studies used populations hospitalized for a mental health condition. All the studies only used Reiki as the treatment under study although in varying dosages and with practitioners who had varying levels of training and experience. A number of studies applied Reiki to populations that were healthy or unhealthy but with normal (clinically irrelevant) levels of anxiety, stress, or depression. As mentioned, this was thought to influence outcomes, so studies were grouped according to normal vs. clinically relevant scores. In this way, consistency was improved, and all showed expected trends. On the whole, despite some variations, it was determined that there was enough consistency not to decrease the level of GRADE ranking.

All the studies met the criteria for *indirectness*; that is, all clearly met PICO inclusion criteria and measured what they were meant to measure. For the criteria of *imprecision*, the small sample sizes across most of the studies would have increased imprecision, decreasing the GRADE down by one level to a grade of *Moderate*. For *publication bias*, while the small sample sizes across most of the studies would have increased publication bias, the systematic search across multiple databases would have eliminated or reduced this bias, so the GRADE assessment was not decreased. This leaves all areas at a GRADE level of moderate because of the criteria of imprecision. However, GRADE can also increase one level when there are large effects or when there are dose-response relationships (Schünemann, 2022; Siemieniuk, 2022), and this will now be assessed.

### Anxiety GRADE

The three RPCTs with populations meeting clinical cutoffs for anxiety produced SOME highly significant results for Reiki’s effectiveness over placebo (*p* = 0.003, *p* = 0.004, and *p* < 0.05), or as has been noted by Baldwin et al. (2017) for Reiki post-treatment (*p* = 0.0001) and not placebo post-treatment. Where they could be calculated, the effect sizes for Reiki, compared with placebo, in two studies ranged from large to very large (*d* = 0.93, 1.36, and 1.07). However, because Baldwin et al. (2017) treatment and placebo groups were not directly compared, GRADE was only increased to a level of moderate to high.

The three studies with normal anxiety scores all produced non-significant results for Reiki’s influence compared with placebo. Here, compared with placebo, the overall GRADE ranking for Reiki in reducing normal anxiety was low.

### Stress GRADE

In the three RPCTs using populations with clinically relevant stress scores, the findings were all highly significant for Reiki compared with placebo (*p* = 0.028, 0.008, and 0.001). They also all produced large or very large effect sizes (*d* = 0.97, 0.9, and 2.3) except for Vasudev and Shastri (2016), which produced a moderate effect (*d* = 0.63). Overall, compared with placebo, this would increase the GRADE to a level of high for Reiki’s influence on reducing clinically relevant levels of stress.

For the two RPCTs on stress with outcomes in the normal range, one (Shore, 2004) found significant results for Reiki over placebo post-treatment (*p* = 0.029) and at 1-year follow-up (*p* = 0.001). As previously noted, this follow-up score should be treated with caution because of high dropouts. Shore’s (2004) study also found large effect sizes post-treatment (*d* = 0.88) and very large effect sizes at follow-up (*d* = 2.02). While the participants did not meet the cutoffs for clinical stress in this study, they met the cutoffs for depression and reported being anxious and stressed. It may be that the PSS-10 did not capture their stress levels adequately or that Reiki, compared with placebo, is also effective at reducing normal levels of stress. These outcomes suggest a low to moderate level of evidence that Reiki is more effective than placebo in reducing stress in the normal range.

### Depression GRADE

For the area of depression, in three of four studies using clinically relevant scores, all produced statistically significant results for Reiki over placebo. The RPCT that did not produce significant outcomes had a high ROB, and as such, the outcomes were likely compromised. For the other three RPCTs with significant outcomes, effect sizes could be calculated in two studies (Dressen and Singg, 1998; Shore, 2004), and both showed some very large (*d* = 1.43 and 1.4) as well as moderate effects (*d* = 0.74). The other study (Erdogan and Cinar, 2016) provided insufficient data for the effects to be calculated but showed very high levels of significance across all the time points including follow-up (*p* = 0.001 to *p* = 0). Because of the large effects and consistent significant findings for Reiki, the GRADE level was increased by one. It was concluded that, compared with placebo, there was a high level of evidence for the influence of Reiki on reducing clinically relevant levels of depression.

The two other depression studies with clinically irrelevant scores produced no significant effects for Reiki when compared with placebo. As such, the GRADE assessment for Reiki reducing normal-range depression when compared with placebo is low.

### Burnout GRADE

Reiki showed significant results when compared with placebo for all the burnout studies. However, they were not highly significant, which may have been because two studies were based on only a single 30-min session of Reiki (Díaz-Rodríguez et al., 2011a,b). It would be interesting to see if the significance increased with a more extended session of Reiki and/or multiple applications and whether the effects of Reiki lasted at follow-up, which were not assessed. There were insufficient data to calculate effect sizes for Rosada et al. (2015) study, and the effect sizes for Díaz-Rodríguez et al. (2011a,b) two studies were mostly moderate (*d* = 0.59, 0.75, and 0.71) to large (*d* = 0.85). These effect sizes were not sufficient to increase the GRADE ranking. Also, as noted earlier, there is some contention as to whether biomarkers adequately operationalize burnout. For this reason, the level of evidence for burnout was downgraded overall to low to moderate. A summary of findings for the reviewed RPCTs along with levels of significance, effect sizes, and ROB 2 and GRADE assessments are presented in Table 1.

## Discussion

To answer to the first research question, to date, the evidence suggests that, compared with placebo, Reiki consistently demonstrates a therapeutic effect on some symptoms of mental health. When Reiki is applied to people with clinically relevant levels of mental health, the GRADE level of evidence is moderate to high for anxiety and high for stress and depression in reducing symptoms over placebo. For people with stress levels in the normal range, the GRADE level of evidence was low to moderate in reducing stress when compared with placebo. When Reiki was applied to people with anxiety and depression in the normal range, the GRADE level of evidence was low for reducing anxiety or depression when compared with over placebo. When Reiki was applied to people with burnout, the GRADE level of evidence was low to moderate in reducing burnout.

Overall, the number of studies in each area is small, and further research is required to confirm the conclusions. A detailed discussion on how well the placebo effect was controlled for by the studies in this review will now be explored. This is followed by a discussion on the second research question; what parameters other than the level of wellness might influence the effectiveness of Reiki over placebo?

### Controlling for Placebo

The placebo effect is powerful and has the ability to alter our biology and enhance our mood (Hamilton, 2021). According to Benson and Friedman (1996), three factors contribute to the placebo effect:

Criterion 1. Placebo can increase positive expectations of the client and influence their beliefs and/or biology to produce the real effect under study (Benson and Friedman, 1996; Hamilton, 2021).

Criterion 2. Placebo can affect the expectations of the practitioner, which in turn influences the expectations of the client to produce the real effect under study (Benson and Friedman, 1996).

Criterion 3. The strength of the relationship between the practitioner and the client can influence client expectations in such a way as to produce the effect under study (Benson and Friedman, 1996).

How well each of these factors was controlled in the studies under review will now be discussed.

Criterion 1: Participant expectations.

All RPCTs in this systematic review met Criterion 1, in that they controlled for participants’ expectations. In three RPCTs, the blinding of participants was also tested and found to be successful (Bowden et al., 2010, 2011; Baldwin et al., 2017).

Criterion 2: Therapist expectations.

Two RPCTs additionally controlled for therapist expectations (Shiflett et al., 2002; Erdogan and Cinar, 2016). To achieve this, the practitioner must believe that they are administering the treatment of choice but really provide a treatment that cannot produce an effect. In Erdogan and Cinar’s (2016) study on elderly depressed patients, the sham practitioners were four nurses who did not have training in Reiki but were made to believe they were practicing Reiki. Details about how the blinding was achieved were not provided. The study produced highly significant long-term effects of Reiki when compared with placebo on treating depression in the elderly. Shiflett et al.’s (2002) study on the effect of Reiki when compared with placebo on functional recovery and depression in stroke victims also blinded a group of sham Reiki practitioners and found no significant effects of Reiki compared with placebo. They blinded the novice practitioners by telling them that they may or may not be attuned to Reiki and then only attuned half of the group of novice practitioners while pretending to attune the other half. This blinding was assessed as successful, but the success of attunement was questionable because it was done at a distance. As discussed, the study had other significant methodological issues resulting in high ROB.

Criterion 3: Strength of the relationship.

The strength and quality of the relationship can also influence outcomes, and this is commonly reported to be an important therapeutic influence in psychotherapy sessions (Flückiger et al., 2018). In the Reiki sessions under study, several factors would have reduced this kind of placebo influence. In all the RPCTs, the therapists and the clients were not allowed to speak or speaking was kept to a minimum. Therefore, verbal cues would have been minimal or non-existent. In some of the RPCTs, sensory cues were further minimized. In Bowden et al.’s (2010, 2011) studies, there were no sensory cues, i.e., the client was blindfolded while the therapist stood behind them and did not touch them or speak. Both studies by Díaz-Rodríguez et al. (2011a,b) on Reiki’s effect on burnout did not allow any touch. Dressen and Singg (1998) study only allowed touch for the head positions but not the body. Vasudev and Shastri’s (2016) study found hands-on Reiki to be significantly more effective than distant placebo Reiki (*p* = 0.028). However, the hands-on Reiki was not significantly different from the distant Reiki (*p* = 0.878), which used no sensory cues at all. This suggests a placebo effect from relationship factors should not have influenced outcomes. Relationship expectations influencing the clients would also have been removed or reduced in the two discussed RPCTs controlling for Criterion 2 (Shiflett et al., 2002; Erdogan and Cinar, 2016). These controlled for therapist expectations, which are central to the relationship.

In sum, all the RPCTs controlled for Criterion 3 to some extent, and eight RPCTs controlled for it to a larger extent (Dressen and Singg, 1998; Shiflett et al., 2002; Bowden et al., 2010, 2011; Díaz-Rodríguez et al., 2011a,b; Erdogan and Cinar, 2016; Vasudev and Shastri, 2016). Of these eight, six showed significant therapeutic outcomes for Reiki over placebo. Erdogan and Cinar’s (2016) study controlled for Criteria 1, 2, and 3 and found highly significant effects for Reiki over placebo, although it still had an ROB of some concerns and did not describe the blinding procedure applied to the therapists.

Hamilton (2021) argues that Reiki’s effects are essentially placebo by-products of criteria B and C, that is, the expectations of the practitioners and the strength of the relationship. He argues that the recipient becomes aware of the emotional states of the real Reiki therapist through their facial expressions and body language and that these are more convincing than the sham Reiki therapist. He also argues that mirror neurons in the brain, which make us mimic the therapist’s emotional states, facilitate this emotional transference, which is enough to produce a statistically significant impact of Reiki from a real therapist over sham Reiki (Hamilton, 2021). This argument is not convincing given the above discussion. As mentioned, in four RPCTs, the participants were blindfolded and/or not touched by the therapists to minimize or eliminate sensory cues. In two other studies, practitioner expectations were controlled for, one of which produced highly significant results.

Hamilton (2021) also argues that the therapist’s emotional state affects their bioelectric current and magnetic field, which has been shown to have the ability to influence another’s bioelectric and magnetic fields. If this is correct, even without visual or tactile cues, a real Reiki’s practitioner’s positive intention may be what is influencing the client over sham Reiki practitioners who have no genuine healing intention toward the client. This possibility is worth considering. However, Erdogan and Cinar’s (2016) RPCT provides evidence to the contrary. In their study, the sham Reiki therapists were made to believe that they were practicing real Reiki, which, according to Hamilton (2021), would have therapeutically enhanced their bioelectric field and magnetic current, thereby influencing the clients’ bioelectric field and magnetic current as much as the real Reiki practitioners did. However, the real Reiki practitioners still produced a therapeutic effect that was highly statistically significant over the sham practitioners despite having the same therapeutic expectations. More well-controlled double-blind experiments are needed to further verify this result. Such studies will also help isolate underlying mechanisms of influence for Reiki. As mentioned at the start of this article, studies that best control for all the placebo Criteria A, B, and C are studies on Reiki’s influence on non-human living systems: cell cultures, isolated cells, rats, and dogs, and all found highly significant results compared with placebo (Baldwin and Schwartz, 2006; Baldwin et al., 2008; Mothersill et al., 2013; Kent et al., 2020; Pacheco et al., 2021).

### Under What Parameters Is Reiki Effective Over Placebo?

#### Clinically Relevant Baseline Scores

As discussed, the findings suggest that Reiki is more effective than placebo when the baseline scores are clinically relevant even if low. There may be other variables impacting energy healing (Griffin and Erdreich, 1991; Oschman, 2015), such as the size of a client’s biofield or the environment in which it is practiced; however, this SR highlights the importance of clinically relevant baselines.

#### Reiki Dosage

The RPCT perhaps showing the most benefit for Reiki in reducing depression was by Erdogan and Cinar (2016), in which Reiki was administered for a longer period of 45 to 60 min one time a week for 8 weeks. However, Shore (2004) also administered Reiki for long periods of 60–90 min one time a week for 6 weeks but achieved moderate significance at post-test (*p* = 0.042). The three RPCTs that produced non-significant results for depression also applied Reiki 6 to 10 times, although for only 20 to 30 min per application (Shiflett et al., 2002; Bowden et al., 2010, 2011). Overall, these RPCTs suggest that 60 min of Reiki for 6 to 10 weeks may be sufficient to produce a significant therapeutic effect for at least mild clinical depression over placebo with potentially long-lasting effects.

For anxiety, the three RPCTs with significant findings all administered 30 min of Reiki on three or more occasions (Dressen and Singg, 1998; Baldwin et al., 2017; Çinar et al., 2022), suggesting that at least four applications of at least 30 min of Reiki one time a week may reduce anxiety compared with placebo.

For stress, Yüce and Taşcı (2021) (2021) administered 45 min of Reiki one time a week for 6 weeks, producing highly significant results in reducing stress when compared with placebo (*p* = 0.001). Bowden et al. (2011) used six applications of 30 min of Reiki again, resulting in highly significant results for stress when compared with placebo (*p* = 0.008). Vasudev and Shastri (2016), however, used very short applications of 5 min of Reiki per day but for 21 consecutive days, producing significant results (*p* = 0.028) although not as high as the other studies using longer treatment periods. Interestingly, Shore (2004), whose participants had normal baseline scores for stress, also produced significant results (*p* = 0.029). Shore (2004) used 60–90 min of Reiki for over 6 weeks, and it may have been the longer treatment period that produced significant results even in participants with normal levels of stress. Overall, compared with placebo, at least six sessions of at least 30 to 45 min of Reiki may reduce stress with potentially long-term effects.

For health professionals diagnosed with burnout, two studies found that a single 30-min session of Reiki can significantly reduce biomarkers related to burnout in the short term (Díaz-Rodríguez et al., 2011a,b). Another study by Rosada et al. (2015) found that 30 min of Reiki one time a week for 6 weeks also significantly reduced burnout when compared with placebo. These studies suggest that one 30-min session of Reiki may benefit the biomarkers of those experiencing burnout in the short term.

#### Type and Level of Training and Experience

Many Reiki practitioners consider that the level of training and years of experience increase the flow of Reiki energy. Some consider the type of Reiki training and how they were trained as also important and advocate the original Usui method through face-to-face training as preferred.

Of the 14 RPCTs, five used Reiki practitioners trained with the original Usui method and of these, two studies stated that they used a practitioner trained solely in the Usui method (Díaz-Rodríguez et al., 2011a; Yüce and Taşcı, 2021). The other three RPCTs stated that they used practitioners trained in the Usui method and one or more in other healing methods (Shiflett et al., 2002; Bowden et al., 2010, 2011). The remaining nine studies only state that the practitioners used Reiki. Given the variation, it is difficult to discern the impact of this variable.

Level of attunement and years of experience are also considered important. Of the 14 RPCTs assessed, six stated they used Reiki masters only (Thornton, 1991; Bowden et al., 2010, 2011; Díaz-Rodríguez et al., 2011a,b; Erdogan and Cinar, 2016). Another three used Reiki masters in addition to either level 2 practitioners (Shore, 2004; Rosada et al., 2015) or level 1 and 2 practitioners (Shiflett et al., 2002). One study used only a level 2 practitioner (Yüce and Taşcı, 2021) and produced highly significant results, suggesting level 2 training may be sufficient for highly significant outcomes. Six studies stated their practitioners’ years of experience (Thornton, 1991; Shore, 2004; Bowden et al., 2010, 2011; Díaz-Rodríguez et al., 2011a,b), ranging from at least 1 year (Shore, 2004) to 15 years (Díaz-Rodríguez et al., 2011a,b). From these outcomes, we might conclude that an experienced level 2 practitioner or a Reiki master with at least 4 years of experience could be used for research purposes.

### Challenges and Limitations of the Evidence

All the areas under review had few studies, so the findings are not conclusive. Several confounding variables other than the level of wellness may also influence outcomes, such as dose, level and type of training, years of practitioner experience, and whether the practitioner was trained in other methods of energy healing and had been trained in ways which may not be effective such as being attuned at a distance. Some of the studies also did not blind the assessors or make it clear whether the outcome assessors were blinded, potentially biasing the outcomes.

### Conclusions

In the area of mental health, so far, there is some evidence that Reiki is consistently more effective than placebo in reducing clinical symptoms of depression, anxiety, stress, and burnout. This effect is observed by decreased symptoms, as measured by validated outcome measures, or validated instruments in each of the areas under study.

So far the research suggests that the duration and frequency of Reiki required to obtain a therapeutic effect over placebo is as follows:

Depression (at least clinically mild): 60 min of Reiki once a week for 6 to 10 weeks with potentially long-term effects lasting from 1 month to 1 year post-treatment.

Anxiety (at least clinically mild): 30 min of Reiki one time a week for a minimum of 4 weeks.

Stress (at least clinically mild): 30 to 45 min of Reiki one time a week for 6 weeks. No long-term effects have been studied.

Stress (normal range): One study showed (Shore, 2004) that 60 to 90 min of Reiki one time a week for over 6 weeks may also reduce normal levels of stress. Effects may be maintained at 1-year follow-up.

Burnout: a single 30-min application of Reiki may reduce some biomarkers of burnout. One study found (Rosada et al., 2015) that six 30-min sessions of Reiki one time a week for 6 weeks may reduce the psychological symptoms of burnout.

For clinical levels of anxiety, stress, and especially depression, a longer duration of treatment and higher number of applications broadly produced more significant outcomes and larger effects. However, low number of studies and other confounders make dose-response relationships unclear. The level of wellness could not be ascertained in one burnout study (Rosada et al., 2015), so dose-response relationships could not be assessed for burnout. All the studies compared the effects of Reiki immediately before and after treatment, suggesting the effects were occurring during the treatment, and sometimes these effects were still strong if measured at follow-up, suggesting that they were maintained or may have continued.

### Recommendations

Future studies controlling for placebo are needed to confirm and consolidate the above findings and should consider the following:

(1) Clinically relevant baseline scores should be used or controlled for and validated outcome measures should be used.
(2) The number or applications of and duration of treatment should be controlled for to establish clearer dosage effects. This can be guided by the above findings.

(3) Long-term effects through follow-up measures are needed to assess the impact of higher doses and lasting impacts.
(4) Outcome measures should be used at several time points to assess the cumulative effects of treatment.
(5) Practitioners should have at least 4 years of practice experience at level two or three to promote therapeutic effects.
(6) To promote consistency, only practitioners trained solely in the original Usui method who have been attuned with hands-on Reiki should be employed.
(7) Studies should always blind the data collectors and assessors.
(8) Blinding of practitioners is ideal and may be conducted by sham attunement vs. real attunement (hands-on). Practitioners should be attuned to level 2 to maximize the effects and the method of blinding outlined in detail.
(9) Where practitioners are not blinded, all sensory and interactive stimuli should be minimized by not allowing any form of communication between the practitioner and the receiver, blindfolding the receiver, and conducting Reiki without touching the body.
(10) Placebo Reiki should not be done by a Reiki practitioner, as Reiki energy may flow without conscious intention.

Controlling for placebo is an important part of investigation in the emerging field of biofield science, and biofield therapies could open up consumers to less expensive, safe, and much-needed treatments in a variety of areas. The findings from this SR provide some evidence for Reiki’s effectiveness over placebo in treating a variety of mental health symptoms, particularly when the symptoms are clinically relevant. Specific parameters for optimal therapeutic effects are unclear; however, the findings so far may be used as a guide. Incorporating Reiki as a complementary treatment to mainstream psychotherapy for depression, stress, and anxiety may be appropriate.

## Data Availability Statement

The datasets presented in this study of full ROB 2 assessments for each study are available at https://cloudstor.aarnet.edu.au/plus/s/qI4RRkw4Ys8MFKr and effect size calculations at https://cloudstor.aarnet.edu.au/plus/s/uCKeNLEjGLo5jId.

## Author Contributions

SZ wrote this manuscript as part of her Ph.D. in Complementary Medicine and Reiki at Bond University. PS is her Ph.D. supervisor and second author of this manuscript. Both authors contributed to the article and approved the submitted version.

## Conflict of Interest

The authors declare that the research was conducted in the absence of any commercial or financial relationships that could be construed as a potential conflict of interest.

## Publisher’s Note

All claims expressed in this article are solely those of the authors and do not necessarily represent those of their affiliated organizations, or those of the publisher, the editors and the reviewers. Any product that may be evaluated in this article, or claim that may be made by its manufacturer, is not guaranteed or endorsed by the publisher.
