# Supplementary material for: Does Reiki Benefit Mental Health Symptoms Above Placebo?
Source: Front Psychol. 2022 Jul 12;13:897312. doi: 10.3389/fpsyg.2022.897312 (PMC9326483; doi:10.3389/fpsyg.2022.897312)
Supplement: Supplementary file 1 [file Image_1.pdf]

Supplementary PRISMA Diagram 1: Flow Chart for Systematic Review

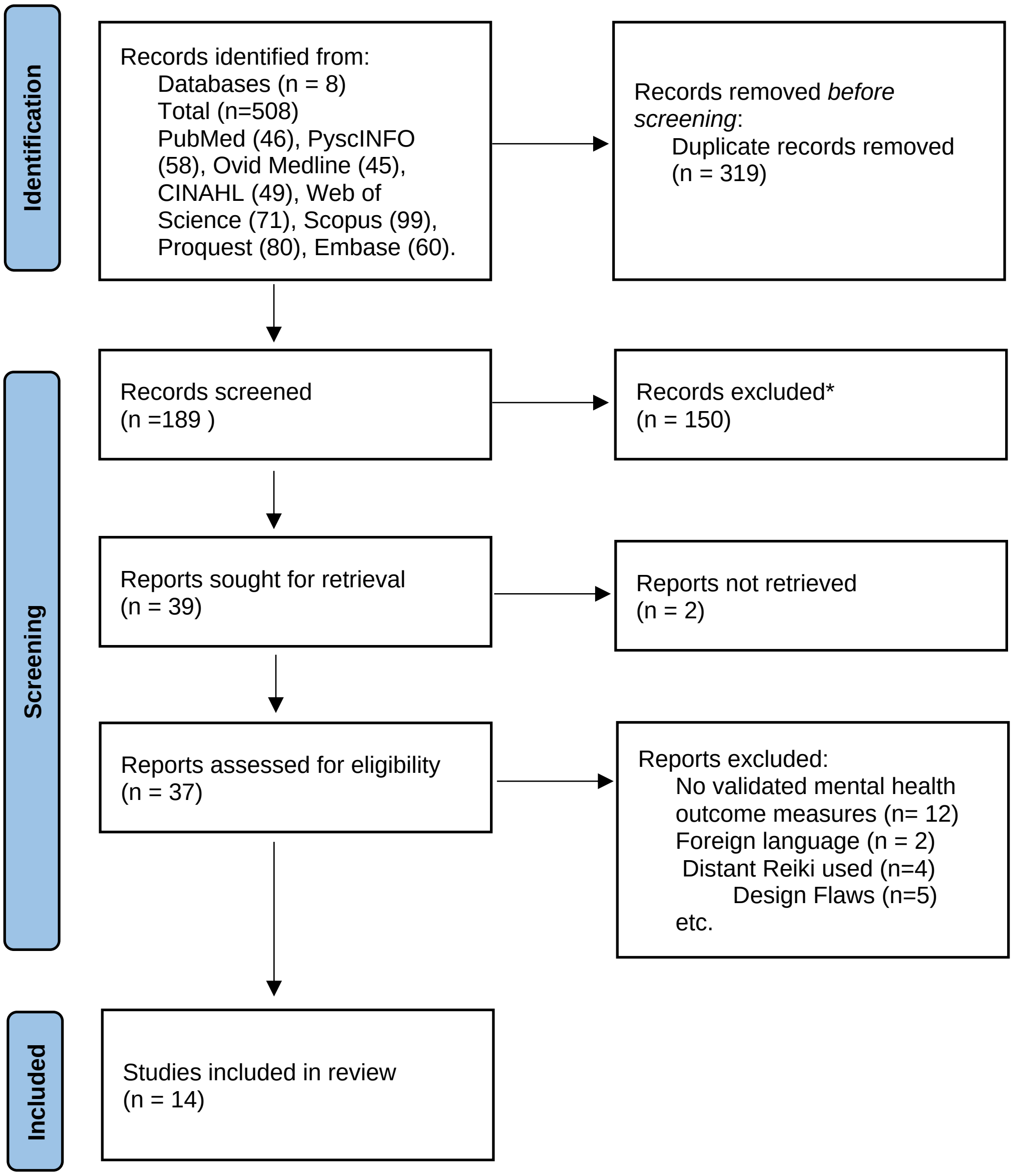

*All excluded by a manually using excel.
