# Supplementary material for: Does Reiki Benefit Mental Health Symptoms Above Placebo?
Source: Front Psychol. 2022 Jul 12;13:897312. doi: 10.3389/fpsyg.2022.897312 (PMC9326483; doi:10.3389/fpsyg.2022.897312)
Supplement: Supplementary file 2 [file Table_1.pdf]

Supplementary Table 1: Extended Summary of Findings

The Effect of Reiki Versus Placebo in Adults
RPCT which used standardized outcome measures for symptoms of Mental Health

Anxiety: Outcomes clinically relevant.
GRADE: HIGH for reducing clinical anxiety

	**Cinar et. al. (2022) Fibromyalgia**	**Baldwin, et al. (2017) Knee Replacement Surgery**	**Dressen & Singg (1998) Chronically ill & in pain.**
**Population**	University Medical Faculty Hospital fibromyalgia.<br>Bursa, Turkey	Acute care hospital sched knee replacem surgery.<br>Arizona, US	Self-selected or GP referred for chronic pain. Texas, US
**N**	50	46	120
**Delivery**	Reiki Hands on<br>Reiki Sham	Reiki Hands on.<br>Sham<br>Quiet time control.	Reiki hands on.<br>PMR<br>Sham<br>Control-quiet reading N=30
**Time x Freq**	30 mins/Wk X 4 weeks.	30min x 3 or 4.	30min x 10 bi-weekly.
**Measure**	STAI	STAI	STAI
**Practitioner Level/Exp**	Reiki by “a researcher” NI re experience or level.	Lev 3 master<br>Not done by researchers.	3 Reiki masters<br>Not done by researcher.
**SS eyes/ touch**	NI re eyes.<br>Touched.	Touched.<br>NI re eyes.<br>Tested blinding after trt and effective.	Touch for head positions held over body for torso. NI re eyes.
**Baseline clinically relevant**	Yes STAI means> 39-40.<br>State 40.18, Trait 51.44	YES overall.<br>R39.1<br>SR42.2<br>C42.6	Anxiety Yes all>39-40 on STAI
**% Females**	88%	Not given. M/F	60
**Significance (P Value)**	**Post 4th trt Reiki sig lower than placebo for state anxiety (p=0.005)<br>And trait anxiety (p=0.003).**	No comparison made due to small sample size of control.<br>**Within grp Reiki sig p=0.004 and Placebo NS.**	**Sig pre-post<br>State Anxiety p=0.0001<br>Trait Anxiety p=0.0001 And Reiki significantly > Placebo (no p given)**
**ROB 2**	Some concerns	Some Concerns	Some Concerns
**Critique**	No pre-specified analysis plan found.<br>--Reiki done by “an experimenter” unclear if an author.	-If the Reiki size better matched placebo Reiki may not have performed as well.<br>-Blinding assessed as successful.<br>-No pre-specified analysis plan found.	-Unstated if assessors blind.<br>-Subjects self-selected in response to advertisement.<br>-No pre-specified analysis plan.<br>-Follow up Reiki scores were not compared to other groups.

Anxiety: outcomes for the normal range.
GRADE: MODERATE for Not Reducing Normal anxiety.

	**Bowden, Goddard and Gruzelier (2010) Well-being.**	**Bowden et al. (2011) Mood and wellbeing in High vs Low Mood**	**Thornton (1991) Female Nurses Unpublished**
**Population**	University students. London UK	University students.<br>London UK	Healthy female volunteer nursing students. California US
**N**	R18 PR17 (n=5 or 6 subgrp)	40 R20 P20 (10 per high and Low)	42 i.e. R22 SR 20
**Delivery**	Non-contact Reiki<br>No reiki (blind 3-30 inches above head)<br>All self-hypnosis/ +ve imagery	Non-contact Reiki (3-30 inches above head)<br>No Reiki	Hands on Reiki<br>Sham R
**Time x Freq**	10 x 20 mins (varied weeks)	30mins x 6 (varied weeks)	1 x 1hr
**Measure**	DASS, HADS.	DASS, HADS.	STAI
**Practitioner Level/Exp**	Lvl 3 Master Usui, Seichim, Violet flame, Ascension R. No yrs exp given.<br>Not Researcher.	Lvl 3 Master Usui,<br>Seichim, Violet flame, Ascension R. 4yrs exper.<br>Not Researcher.	Author administered Reiki 10 year’s experience.
**SS eyes/ touch**	No touch 3-30 inches above head/back.<br>Blindfolded.	No touch 3-30 inches above head/back.<br>Blindfolded	“Hands on” assumed. NI re eyes.
**Baseline clinically relevant**	No all healthy psych undergraduates.	No Anx. Borderline normal cutoff 7. HADS<br>Mild/mod cut off. Sep means not given.	No acute/ chronic cond.<br>All below cut-off 39-40 on STAI.
**% Females**	60%	82	100%
**Significance (P Value)**	Anxiety subscale DASS p=0.295	Anxiety subscale DASS p=0.084 (high mood improvements maint f.up, placebo anxiety increased).	State Anxiety p=0.864<br>Trait Anxiety p=0.350
**ROB 2**	Some Concerns	Some Concerns	Some Concerns

**Critique**	-Author administered Reiki but no touch (blinding successful p<0.05)<br>-Very small sample size likely decreased power (5 or 6 in each group)<br>-No pre-specified plan.<br>-Dropouts 14%<br>-High variation in time delivered but controlled.	-Very small sample decreased power.<br>-Experimenter administered Reiki however well blinded and participant blinding assessed as successful.<br>-High variation in time delivered but controlled.	- Small sample decreased power.<br>-Assessors were not blind<br>-Experimenter administered Reiki.<br>-Baseline differences not statistically analysed.

Stress: Outcomes Clinically Relevant.
GRADE: HIGH for reducing clinical stress.

	**Bowden et al. (2011) Mood and wellbeing in High vs Low Anxiety and Depression.**	**Vasudev & Shastri (2016) Self-perceived work-related stress software professionals.**	**Yuce and Tasci (2021) Stressed Caregivers of cancer patients.**
**Population**	University students.<br>London UK	Software proff from IT companies complaining of high stress.<br>Bangalore India.	Oncology unit of a university hospital, Turkey.
**N**	40 R20 P20 (10 per high and Low)	120	42<br>R21<br>S21
**Delivery**	Non-contact Reiki<br>No Reiki	Hands on Reiki<br>Distant Placebo<br>No Reiki.	Hands on Reiki<br>Sham R
**Time x Freq**	30mins x 6 Varied wks	5 mins x 21 over 21 days.	45mins/wk for 6 weeks.
**Measure**	DASS	Perceived Stress Scale (PSS)	Caregiver Strain Index<br>(CSI) 7 or > =high stress.
**Practitioner Level/Exp**	Lvl 3 Master Usui, Seichim, Violet flame, Ascension R. 4yrs experience.<br>Not Researcher.	Unstated level.<br>Author trained in Usui<br>Reiki done by researcher.	Lvl 2<br>Investigator Usui<br>Experience: NI
**SS eyes/ touch**	No touch 3-30 inches above head/back.<br>Blindfolded	Assume touch "hands on" and NI eyes.	Touching 9 points. NI re eyes.
**Baseline clinically relevant**	Yes Stress Mild range (11.2 in high mood) 6 cut off.	Yes All scores Mod- HIGH. For Stress.	Yes all high stress >7 on CSI.
**% Females**	82	Not given	100%
**Significance (P Value)**	**R Sig reduced stress over > placebo p=0.008.**	**Hands on Reiki and DR Placebo p=0.028**<br>**DR and DRP**<br>**p= 0.019**<br>No diff bet hands on Reiki and DR p=0.878 suggests benefits from hands on R not due to touch or other placebo effects.	**Sig**<br>**CSI p<0.001 at post-treatment 6 weeks.**
**ROB 2**	Some Concerns	Some Concerns	Some Concerns
**Critique**	-Very small sample decreased power.<br>-Experimenter administered Reiki however well blinded and participant blinding assessed as successful.<br>-High variation in time delivered but controlled.	-Hands on and distant Reiki delivered by the experimenter but placebo told were given Reiki, and distant reiki told given placebo reiki.<br>No diff bet dist reiki and hands on reiki suggests hands on not influence by placebo effect.<br>-31% dropout rate (raw scores in thesis) though reasons mostly random.	Investigator administered Reiki and trained sham group.<br>-17% dropouts and no ITT analysis reported.

Stress: Outcomes Normal
GRADE: LOW to MODERATE for reducing normal stress.

	**Bowden, Goddard and Gruzelier (2010) Well-being.**	**Shore (2004) Depression and Self Perceived Stress.**
**Population**	University students. London UK	Self-selected resp to ad. need trt for dep & stress,

		California, U.S.
**N**	R18 PR17	46
**Delivery**	Non-contact Reiki<br>No reiki (blind)<br>All self-hypnosis/ +ve imagery	Reiki, Distant Reiki or distant Reiki placebo
**Time x Freq**	10 x 20 mins	60-90 min/once wk for 6 wks.
**Measure**	HADS, DASS	PSS-10
**Practitioner Level/Exp**	Lvl 3 Master Usui, Seichim, Violet flame, Ascension R. No yrs exp given.<br>Not Researcher.	12 Reiki masters, 3 level 2 practitioners. No researchers.
**SS eyes/ touch**	No touch 3-30 inches above head/back.<br>Blindfolded.	"Hands on" assumed. NI re eyes.
**Baseline Clin. Relevant.**	No healthy psych undergraduates.	Scores PSS v low yet pre-screened for sig anxiety and depression
**% Females**	60%	Not given.
**Significance (P Value)**	NS (p=0.054) stress subscale over placebo on DASS.	Post-test p=0.029<br>One year follow up p =0.001
**ROB 2**	Some Concerns	Some Concerns
**Critique**	Author administered Reiki but no touch (blinding successful p<0.05)<br>-Very small sample size likely decreased power (5 or 6 in each group)<br>-No pre-specified plan.<br>-Dropouts 14%	High drop out from post treatment to f. up though analyzed on original numbers. (73 to 46)<br>-Placebo R led to believe Hands On was the placebo grp to reduce expectancy.<br>-SS self-selected in response to advertisement as being in need of treatment for self-perceived depression and stress/anxiety.

Depression: Outcomes Clinically Relevant.
GRADE: HIGH for reducing clinical depression

	**Dressen & Singg (1998) Chronically ill & in pain.**	**Shore (2004) Depression and Self Perceived Stress.**	**Shiflett et. al. (2002) Functional Recovery Post Stroke Rehab.**	**Erdogan & Cinar (2016) Depression in the Elderly.**
**Population**	Self selected or GP refered for chronic pain.<br>Texas, US	Self-selected resp to ad. need trt for dep & stress, California, U.S.	Kessler Institute for Rehabilitation.<br>US	2 nursing homes<br>Instanbul Turkey
**N**	120	46	50 total<br>10RMaster<br>10RNovice<br>10Sham<br>20 historic control	90<br>R30<br>S30<br>C30
**Delivery**	Reiki hands on.<br>PMR<br>Sham<br>Control-quiet reading<br>N=30	Reiki, Distant Reiki or distant Reiki placebo.	Hands On	Reiki<br>Sham<br>At rest control<br>Hands on.
**Time x Freq**	30min x 10 bi-weekly.	60-90min/once wk for 6 wks.	6-10 x 30mins varied weeks.	45-60mins/ once wk for 8 weeks. 5 mins/ position
**Measure**	STAI	PSS-10	CES-D<br>FIM	DASS<br>Geriatric Depression Scale long. Measured 1st, 4th, 8th week and 1 month f up at 12 weeks.
**Practitioner Level/Exp**	3 Reiki masters<br>Not done by researcher.	12 Reiki masters, 3 level 2 practitioners. No researchers.	Lvl 3<br>Master Usui R Alliance<br>>10 yrs exp.<br>& Lvl 1	Lvl 3<br>Master<br>Unstated Exp.<br>Reiki done by researcher.
**SS eyes/ touch**	Touch for head postitions held over body for torso. NI re eyes.	"Hands on" assumed. NI re eyes.	Double blind. Sham Practitioners thought it was real. Hands on. NI re eyes.	Touch yes.NI eyes. Sham pract belived they delivered Reiki.
**Baseline clinically relevant**	Anxiety Yes all>39-40 on STAI	Scores PSS v low yet pre-screened for sig anxiety and depression	Yes<br>Mild Depression.<br>16 cut-off on CES-D<br>All means but one above this but low.	Yes<br>Mod to severe clinical dep.<br>14 or higher on GDS
**% Females**	60	Not given.	42	63.3

**Significance (P Value)**	**Reiki sig>placebo (no p value)<br>P=0.0001 pre-post. d**	Post test p=0.029<br>One year follow up p =0.001	NS<br>Depression (p>0.05).	**Sig at all 4-time measurements.<br>1st p=0.001 4th p=0.000<br>8th p=0.000 12th p=0.000 (1 mth f.up)**
**ROB 2**	Some Concerns	Some Concerns	HIGH	Some Concerns
**Critique**	-Unstated if assessors blind.<br>-Subjects self-selected in response to advertisement.<br>-No pre-specified analysis plan found.<br>-Follow up Reiki scores were not compared to other groups	High drop out from post treatment to f. up though analyzed on original numbers. (73 to 46)<br>-Placebo R led to believe Hands On was the placebo grp to reduce expectancy.<br>-SS self-selected in response to advertisement as being in need of treatment for self-perceived depression and stress/anxiety.	-double blinded i.e. PR thought they may be attuned in Reiki.<br>-20 historic controls used.<br>-16% missing data and cognitive FIM missing so data excluded from analysis. R often shown to influence cognition so > relevant to outcomes. ITT not done.<br>-Sig diff in age and severity of impairment at baseline – these made covariates but still may have affected results.<br>-No pre-specified analysis plan.<br>-Variation of 6-10 trts though not related to group assignment.	-One of only 2 studies blinding sham practitioners i.e. Reiki done by researcher but Placebo Reiki practitioners believed they were doing Reiki to control for practitioner expectancy effects.<br>-NI whether outcome assessors were blind.

Depression: Outcomes Normal
GRADE: MODERATE for NOT reducing normal depression.

	**Bowden, Goddard and Gruzelier (2010) Well-being.**	**Bowden et al. (2011) Mood and wellbeing in High vs Low Anxiety and Depression.**
**Population**	University students. London UK	University students.<br>London UK
**N**	R18 PR17 (n=5 or 6 subgrp)	40 R20 P20 (10 per high and Low)
**Delivery**	Non-contact Reiki<br>No reiki (blind)<br>All self-hypnosis/ +ve imagery	Non-contact Reiki<br>No Reiki
**Time x Freq**	10 x 20 mins varied weeks	30mins x 6 varied weeks
**Measure**	DASS<br>HADS	DASS<br>HADS
**Practitioner Level/Exp**	Lvl 3 Master Usui, Seichim, Violet flame, Ascension R. No yrs exp given.<br>Not Researcher.	Lvl 3 Master Usui,<br>Seichim, Violet flame, Ascension R. 4yrs exper.<br>Not Researcher.
**SS eyes/ touch**	No touch 3-30 inches above head/back.<br>Blindfolded.	No touch 3-30 inches above head/back.<br>Blindfolded
**Baseline clinically relevant**	Healthy psych undergraduates.	No below cut-off for depression.
**% Females**	60%	82
**Significance (P Value)**	NS (p>0.05 no values given).	NS (p>0.05 no values given)
**ROB 2**	Some Concerns	Some Concerns
**Critique**	Author administered Reiki but unlikely to influence as sat behind a blindfolded SS no touch (blinding successful p<0.05)<br>-Very small sample size likely decreased power (5 or 6 in each group)<br>-No pre-specified plan.<br>-Dropouts 14%	Variation in the period of treatment: 2 to 8 weeks doesn't state how variation distributed bet groups.<br>-Very small sample decreased power.<br>-Experimenter administered Reiki.<br>-Participant blinding was assessed as successful.

Burnout
GRADE: MODERATE for reducing burnout.

	Díaz-Rodríguez et al., (2011) Nurses with Burnout.	Díaz-Rodríguez et al., (2011) Health Care Proff with Burnout. –	Rosada et.al. (2015) Burnout Mental health care professional.
**Population**	University hospital diagnosed with burnout. Granada, Spain.	University hospital diagnosed with burnout.<br>Granada, Spain.	Community mental health agencies New England, US
**N**	18	21	45

**Delivery**	Hands not touching over body	Hands not touching over body	Hands on Reiki<br>Sham R
**Time x Freq**	30min x 1	30min x 1	30mins/week for 6 weeks then swap to 30 mins sham/wk for 6 weeks.
**Measure**	Omron HEM-737 validated. Average of Triplicate Measurements used.	OMRON 510 for body temp.<br>Saliva- lumin immune assay.<br>Malach to diagnose burnout.<br>Standardized software HRV and ECG.	Maslach Burnout Inventory<br>Measure your medical outcome profile
**Practitioner Level/Exp**	Lvl 3<br>Master<br>>15 yrs experience.<br>Usui	3 master<br>15 yrs experience	Lvl 2 & 3<br>6 mast 10 lvl 2.<br>5-21 yrs exp.<br>Not Researchers.
**SS eyes/ touch**	No touch “over various parts body”<br>NI eyes.	No touch “over various parts body”<br>NI eyes.	Yes touch.<br>NI re eyes.
**Baseline clinically relevant**	Yes<br>Diagnosed Burnout Syndrome.	Yes<br>Diagnosed Burnout Syndrome.	NI if Burnout met cut-off on MBI means not given.
**% Females**	100	100	73
**Significance**<br>**(P Value)**	**Reiki reduced diastolic BP p=0.04**<br>**Increased SIgA p= 0.04**<br>NS<br>Systolic BP p=0.24<br>a-amylase activity p=0.71	**ECG recordings for SDNN sig higher then PR (p<0.04).**<br>**Sig higher body temp then PR (p=0.02).**<br>**Higher body temp sig corr with LF domain after Reiki (p=0.02) suggesting**<br>**effect on parasympatheticNS**<br>NS over PR<br>Salivary Cortisal (p=0.08)<br>ECG RMSSD (p=0.06).	**Sig**<br>**p=0.011**<br>**Reiki reduced burnout over placebo. Also reduced emotional exhaustion, depersonal, increased pers accomp (p<0.05 no values given)**.
**ROB 2**	Some Concerns	Some Concerns	Some Concerns
**Critique**	NI found on pre-register.	- NI found on pre-register.<br>-blinding of participants tested and found to be successful.	-It is not stated whether volunteer outcome assessors were blind.<br>-Published study excludes some non-significant results found in original thesis suggesting selective reporting.<br>-baseline numbers between groups for single people not given and this is important to some results.

Bolded text indicates significant outcomes.
