# Supplementary material for: Does Reiki Benefit Mental Health Symptoms Above Placebo?
Source: Front Psychol. 2022 Jul 12;13:897312. doi: 10.3389/fpsyg.2022.897312 (PMC9326483; doi:10.3389/fpsyg.2022.897312)
Supplement: Supplementary file 3 [file Data_Sheet_1.pdf]

Supplementary Appendix A: Systematic Review Search Terms

PubMed
reiki[tiab] AND (sham[tiab] OR placebo[tiab] OR mock[tiab] OR comparison[tiab])

Ovid Medline
reiki.tw. AND (sham.tw. OR placebo.tw. OR mock.tw. OR comparison.tw.)

Embase Elsevier
reiki:ti,ab AND (sham:ti,ab OR placebo:ti,ab OR mock:ti,ab OR comparison:ti,ab)

Embase Ovid
reiki.tw. AND (sham.tw. OR placebo.tw. OR mock.tw. OR comparison.tw.)

CINAHL
(TI reiki OR AB reiki) AND ((TI sham OR AB sham) OR (TI placebo OR AB placebo) OR (TI mock OR AB mock) OR (TI comparison OR AB comparison))

Web of Science
reiki AND (sham OR placebo OR mock OR comparison)

SCOPUS
reiki AND (sham OR placebo OR mock OR comparison)

PsychInfo (Ovid)
reiki.ti,ab. AND (sham.ti,ab. OR placebo.ti,ab. OR mock.ti,ab. OR comparison.ti,ab.)

ProQuest Health and Medical
TI,AB(reiki) AND (TI,AB(sham) OR TI,AB(placebo) OR TI,AB(mock) OR TI,AB(comparison))
