# Supplementary material for: Does Reiki Benefit Mental Health Symptoms Above Placebo?
Source: Front Psychol. 2022 Jul 12;13:897312. doi: 10.3389/fpsyg.2022.897312 (PMC9326483; doi:10.3389/fpsyg.2022.897312)
Supplement: Supplementary file 4 [file Data_Sheet_2.pdf]

PRISMA 2020 Checklist

Section and Topic	Item #	Checklist item	Location where item is reported
**TITLE**			
Title	1	Identify the report as a systematic review.	In the Abstract and on p3 at the start of the Method.
**ABSTRACT**			
Abstract	2	See the PRISMA 2020 for Abstracts checklist.	All checks for abstract met.
**INTRODUCTION**			
Rationale	3	Describe the rationale for the review in the context of existing knowledge.	Introduction p2 and p3.
Objectives	4	Provide an explicit statement of the objective(s) or question(s) the review addresses.	Method under Research Questions p4 and p5.
**METHODS**			
Eligibility criteria	5	Specify the inclusion and exclusion criteria for the review and how studies were grouped for the syntheses.	These are stated in PICO in the method section on p4 and in the results on p14 under “Grade Ranking”.
Information sources	6	Specify all databases, registers, websites, organisations, reference lists and other sources searched or consulted to identify studies. Specify the date when each source was last searched or consulted.	PRISMA flowchart p21 and p22 and Search Method p5. The last time each source was searched for or consulted was on 26th March 2022.
Search strategy	7	Present the full search strategies for all databases, registers and websites, including any filters and limits used.	This is found in the PRISMA flowchart on p 21 and p 22 and in the Search Method p5 and in the Appendix A p34 to p35 Systematic Review search terms.
Selection process	8	Specify the methods used to decide whether a study met the inclusion criteria of the review, including how many reviewers screened each record and each report retrieved, whether they worked independently, and if applicable, details of automation tools used in the process.	All studies were screened by the author according to PICO in the Method section on p4 and the second author independently and manually screened each record and report retrieved. See Search Method p5. Appendix A p34 to p35 Systematic Review search terms.
Data collection process	9	Specify the methods used to collect data from reports, including how many reviewers collected data from each report, whether they worked independently, any processes for obtaining or confirming data from study investigators, and if applicable, details of automation tools used in the process.	Data was manually extracted from studies where available and effect sizes were calculated using an effect size calculator then randomly checked calculating Cohen’s d from t-tests. The 2nd author independently reviewed the data and results. See Search Method p 5.
Data items	10a	List and define all outcomes for which data were sought. Specify whether all results that were compatible with each outcome domain in each study were sought (e.g. for all measures, time points, analyses), and if not, the methods used to decide which results to collect.	Data was only sought for effect sizes.
	10b	List and define all other variables for which data were sought (e.g. participant and intervention characteristics, funding sources). Describe any assumptions made about any missing or unclear information.	Baseline means of all groups in all studies were manually compared with clinical cut-offs of the outcome measures used. Studies were grouped where possible into clinically relevant and non-clinically relevant scores and GRADED accordingly. See Results p 6 to 16 and GRADE ranking p 14. Where data was unavailable to calculate effect sizes this was stated in the critique of each study in the results section from pages 6 to 16.
Study risk of bias assessment	11	Specify the methods used to assess risk of bias in the included studies, including details of the tool(s) used, how many reviewers assessed each study and whether they worked independently, and if applicable, details of automation tools used in	The Cochrane ROB 2 assessment tool was used the first author following the given Cochrane algorithm as a guide. After completion the second author independently reviewed all decisions in discussion with the first

Section and Topic	Item #	Checklist item	Location where item is reported
		the process.	author. Discrepancies were discussed and agreed upon. No automation tools were used.
Effect measures	12	Specify for each outcome the effect measure(s) (e.g. risk ratio, mean difference) used in the synthesis or presentation of results.	Mean difference (Placebo) was compared with the mean difference of Reiki group used to calculate effect sizes.
Synthesis methods	13a	Describe the processes used to decide which studies were eligible for each synthesis (e.g. tabulating the study intervention characteristics and comparing against the planned groups for each synthesis (item #5)).	All studies meeting PICO included in SR and those with available data used to calculate effect sizes.
	13b	Describe any methods required to prepare the data for presentation or synthesis, such as handling of missing summary statistics, or data conversions.	Missing effect sizes due to unavailable data were stated as such throughout the results section p 6 to 16.
	13c	Describe any methods used to tabulate or visually display results of individual studies and syntheses.	Table 1: Summary of Main Findings p 22- p24 and Table 2: Extended Summary of Findings in Supplementary Material p 30 – p 34 were used to visually display and synthesise results.
	13d	Describe any methods used to synthesize results and provide a rationale for the choice(s). If meta-analysis was performed, describe the model(s), method(s) to identify the presence and extent of statistical heterogeneity, and software package(s) used.	NA
	13e	Describe any methods used to explore possible causes of heterogeneity among study results (e.g. subgroup analysis, meta-regression).	NA
	13f	Describe any sensitivity analyses conducted to assess robustness of the synthesized results.	NA
Reporting bias assessment	14	Describe any methods used to assess risk of bias due to missing results in a synthesis (arising from reporting biases).	Cochrane ROB 2 algorithm.
Certainty assessment	15	Describe any methods used to assess certainty (or confidence) in the body of evidence for an outcome.	Cochrane ROB 2 tool and GRADE assessment criteria. See results p 6 to 16.
**RESULTS**			
Study selection	16a	Describe the results of the search and selection process, from the number of records identified in the search to the number of studies included in the review, ideally using a flow diagram.	See PRISMA flow chart p 21- p 22.
	16b	Cite studies that might appear to meet the inclusion criteria, but which were excluded, and explain why they were excluded.	These are cited and reasons for exclusion are provided in the search method on p 5.
Study characteristics	17	Cite each included study and present its characteristics.	This is provided in the results section p 6 to 16 as well as in Table 1: Summary of Main Findings p 22- p24 and Table 2: Extended Summary of Findings in Supplementary Material p 30 – p 34.
Risk of bias in studies	18	Present assessments of risk of bias for each included study.	A cloudstor link to full ROB 2 assessments is provided in the method section on p 5. https://cloudstor.aarnet.edu.au/plus/apps/files/?dir=/&fileid=6443424441

Section and Topic	Item #	Checklist item	Location where item is reported
			Final assessments of ROB are also provided in the results section p 6 to 16, Table 1: Summary of Main Findings p 22- p24 and Table 2: Extended Summary of Findings in Supplementary Material p 30 – p 34.
Results of individual studies	19	For all outcomes, present, for each study: (a) summary statistics for each group (where appropriate) and (b) an effect estimate and its precision (e.g. confidence/credible interval), ideally using structured tables or plots.	P values, and effects sizes were discussed for each study in the results section on p 6 to 16 and were also presented in Table 1: Summary of Main Findings p 22- p24.
Results of syntheses	20a	For each synthesis, briefly summarise the characteristics and risk of bias among contributing studies.	This was done in the results section on p 6 to 16 and these were also summarised in Table 1: Summary of Main Findings p 22- p24.
	20b	Present results of all statistical syntheses conducted. If meta-analysis was done, present for each the summary estimate and its precision (e.g. confidence/credible interval) and measures of statistical heterogeneity. If comparing groups, describe the direction of the effect.	See Table 1: Summary of Main Findings for effect sizes p 22-24. Raw data used to calculate effect sizes can be found at: https://cloudstor.aarnet.edu.au/plus/s/uCKeNLEjGLo5jId
	20c	Present results of all investigations of possible causes of heterogeneity among study results.	These were discussed under GRADE Ranking on p 14 and p 15 in the discussion of inconsistency.
	20d	Present results of all sensitivity analyses conducted to assess the robustness of the synthesized results.	NA
Reporting biases	21	Present assessments of risk of bias due to missing results (arising from reporting biases) for each synthesis assessed.	See the cloudstor link to full ROB 2 assessments p 5 to see assessment of missing results.<br>at https://cloudstor.aarnet.edu.au/plus/apps/files/?dir=/&fileid=6443424441
Certainty of evidence	22	Present assessments of certainty (or confidence) in the body of evidence for each outcome assessed.	See results p 6 to 16 and in particular GRADE ranking on p14 to p16. Also see Table 1: Summary of Main Findings for effect sizes p 22 – p24..
**DISCUSSION**			
Discussion	23a	Provide a general interpretation of the results in the context of other evidence.	See the discussion p 16 to p 21.
	23b	Discuss any limitations of the evidence included in the review.	See discussion p 16 to p 21.
	23c	Discuss any limitations of the review processes used.	NA
	23d	Discuss implications of the results for practice, policy, and future research.	See discussion particularly p19 to p21.
**OTHER INFORMATION**			
Registration and protocol	24a	Provide registration information for the review, including register name and registration number, or state that the review was not registered.	My systematic review on placebo only studies of Reiki was registered 29th October 2020 (registration number CRD42020194311).
	24b	Indicate where the review protocol can be accessed, or state that a protocol was not prepared.	Protocol can be accessed by logging in to PROSPERO and accessing https://www.crd.york.ac.uk/PROSPERO
	24c	Describe and explain any amendments to information provided at registration or in the protocol.	The focus area of Reiki studies was broadened to allow for a greater number of studies. The number was then deemed too large and Prospero was contacted about this and they were asked whether more than one review could potentially be published from the included studies overall. They replied on 30/9/21 "The publication of your review is up to you.

PRISMA 2020 Checklist

Section and Topic	Item #	Checklist item	Location where item is reported
			Please don't create a separate protocol though for each."
Support	25	Describe sources of financial or non-financial support for the review, and the role of the funders or sponsors in the review.	NA – there were none.
Competing interests	26	Declare any competing interests of review authors.	None.
Availability of data, code and other materials	27	Report which of the following are publicly available and where they can be found: template data collection forms; data extracted from included studies; data used for all analyses; analytic code; any other materials used in the review.	Links to cloudstor to access full ROB 2 assessments can be found at https://cloudstor.aarnet.edu.au/plus/apps/files/?dir=/&fileid=6443424441<br>To access effect size data https://cloudstor.aarnet.edu.au/plus/s/uCKeNLEjGLo5jId

From: Page MJ, McKenzie JE, Bossuyt PM, Boutron I, Hoffmann TC, Mulrow CD, et al. The PRISMA 2020 statement: an updated guideline for reporting systematic reviews. BMJ 2021;372:n71. doi: 10.1136/bmj.n71

For more information, visit: http://www.prisma-statement.org/
